# Agnathan VIP, PACAP and Their Receptors: Ancestral Origins of Today's Highly Diversified Forms

**DOI:** 10.1371/journal.pone.0044691

**Published:** 2012-09-05

**Authors:** Stephanie Y. L. Ng, Billy K. C. Chow, Jun Kasamatsu, Masanori Kasahara, Leo T. O. Lee

**Affiliations:** 1 School of Biological Sciences, The University of Hong Kong, Hong Kong, Special Administrative Region, China; 2 Department of Pathology, Graduate School of Medicine, Hokkaido University, Kita-ku, Japan; University of Leuven, Belgium

## Abstract

VIP and PACAP are pleiotropic peptides belonging to the secretin superfamily of brain-gut peptides and interact specifically with three receptors (VPAC_1_, PAC_1_ and VPAC_2_) from the class II B G protein-coupled receptor family. There is immense interest regarding their molecular evolution which is often described closely alongside gene and/or genome duplications. Despite the wide array of information available in various vertebrates and one invertebrate the tunicate, their evolutionary origins remain unresolved. Through searches of genome databases and molecular cloning techniques, the first lamprey VIP/PACAP ligands and VPAC receptors are identified from the Japanese lamprey. In addition, two VPAC receptors (VPACa/b) are identified from inshore hagfish and ligands predicted for sea lamprey. Phylogenetic analyses group these molecules into their respective PHI/VIP, PRP/PACAP and VPAC receptor families and show they resemble ancestral forms. Japanese lamprey VIP/PACAP peptides synthesized were tested with the hagfish VPAC receptors. hfVPACa transduces signal via both adenylyl cylase and phospholipase C pathways, whilst hfVPACb was only able to transduce through the calcium pathway. In contrast to the widespread distribution of VIP/PACAP ligands and receptors in many species, the agnathan PACAP and VPAC receptors were found almost exclusively in the brain. *In situ* hybridisation further showed their abundance throughout the brain. The range of VIP/PACAP ligands and receptors found are highly useful, providing a glimpse into the evolutionary events both at the structural and functional levels. Though representative of ancestral forms, the VIP/PACAP ligands in particular have retained high sequence conservation indicating the importance of their functions even early in vertebrate evolution. During these nascent stages, only two VPAC receptors are likely responsible for eliciting functions before evolving later into specific subtypes post-Agnatha. We also propose VIP and PACAP's first functions to predominate in the brain, evolving alongside the central nervous system, subsequently establishing peripheral functions.

## Introduction

Since their initial discoveries, the knowledge in respect to vasoactive intestinal peptide (VIP) and pituitary adenylate cyclase activating polypeptide (PACAP) are no longer limited to their founding species: the hog and ovine, respectively [1], [2]. Instead, a variety of sequences ranging from each vertebrate class are now known. Both VIP and PACAP are pleiotropic hormones belonging to the secretin superfamily of brain-gut peptides, amongst which VIP and PACAP have highest resemblance, sharing up to 68% homology. To elicit their physiological functions, VIP and PACAP bind specifically with three receptors belonging to the class II B G protein-coupled receptors (GPCRs): VPAC_1_, VPAC_2_ and PAC_1_, which upon activation initiate a cascade of transduction signals involving secondary messengers such as cyclic adenosine monophosphate (cAMP) and calcium ions. Of the receptor trio, VPAC_1_ was the first to be cloned from the rat lung cDNA library [3], followed by VPAC_2_ from the rat pituitary cDNA library [4] and PAC_1_ [5]–[10], and now all three receptors from various vertebrate classes. With the vast collection of data, this has led to great interest in revealing the origins and processes in the evolution of VIP/PACAP ligands and receptors.

Based on the structural similarities amongst VIP and PACAP, co-evolution from a common ancestral gene has been proposed [11], [12], beginning with an initial exon duplication followed by gene and/or genome duplications. This has been further supported by findings of duplicate VIP/PACAP copies in teleosts [13], [14], which are suggested to be products of the teleost specific gene duplication event (3R) [15]. Tunicate (*Chelyosoma productum*) PACAP cDNAs [16] have also been reported, suggesting a history of existence dating as early as prior to the protostome-vertebrate split, however the authenticity of these sequences remains a controversy with the failure to identify homologues in available completed genomes [17], [18], [19]. Nevertheless, on the basis of PACAP being more structurally conserved (in terms of length and sequence identity of cDNAs, peptides and genomic environment) than VIP (Figure S1), the ancestral VIP/PACAP gene is believed to be “PACAP-like” before being duplicated to create VIP later in the vertebrate lineage [18]. Likewise, the VIP/PACAP receptors are also proposed to share a mutual primordial gene based on their high sequence identities, with at least 50% homology between any two receptors [3], [4], [8], [17], [20], [21]. Genomic data from various representative vertebrate species has also proved useful in providing some clues regarding the evolutionary history of the VIP/PACAP receptor trio, with VPAC_1_ and PAC_1_ co-localized on one chromosome and VPAC_2_ on another (Figure S2). Based on these findings, it has been suggested that the first duplication event gave rise to two genes, one encoding for VPAC_1_ and PAC_1_, and another for VPAC_2_, and it was a subsequent round of duplication which then separated these receptors into their specific subtypes [21]. To date the collection of information available for VIP/PACAP ligands and receptors is vast; however the scarcity of information with regards to early vertebrates has resulted in the initial evolutionary events remaining enigmatic.

To gain better insights towards these evolutionary episodes, we have characterized VIP/PACAP ligands and receptors from the agnathan fish class. As initial pioneers of the ancestral fish line, the agnathans mark a turning point in evolution giving rise to the first gnathostomes over 550 million years ago (mya). Their evolutionary importance is further implicated with genome duplication events estimated to occur closely with the evolution of this ancient fish line. More specifically, the first genome duplication (1R) is proposed to occur in the ancestral protochordate lineage prior to the emergence of Agnathans, and the second genome duplication (2R) is believed to be either during or after the Agnathan lineage [11], [22], whilst it has also been suggested that both rounds of duplication occurred prior to the cyclostome-gnathostome split [23]. Although fossil records indicate agnathans to be most abundant during the late Silurian and early Devonian periods [24], modern day resources are limited to the lamprey and hagfish as the only two extant members. With the present study focusing on these living descendants, we hope to unravel the nascent status of VIP/PACAP ligand-receptor forms, providing better insights into the early evolutionary events.

## Results

### First identification of agnathan VIP/PACAP peptides in *L.japonicum*

**Figure 1:**
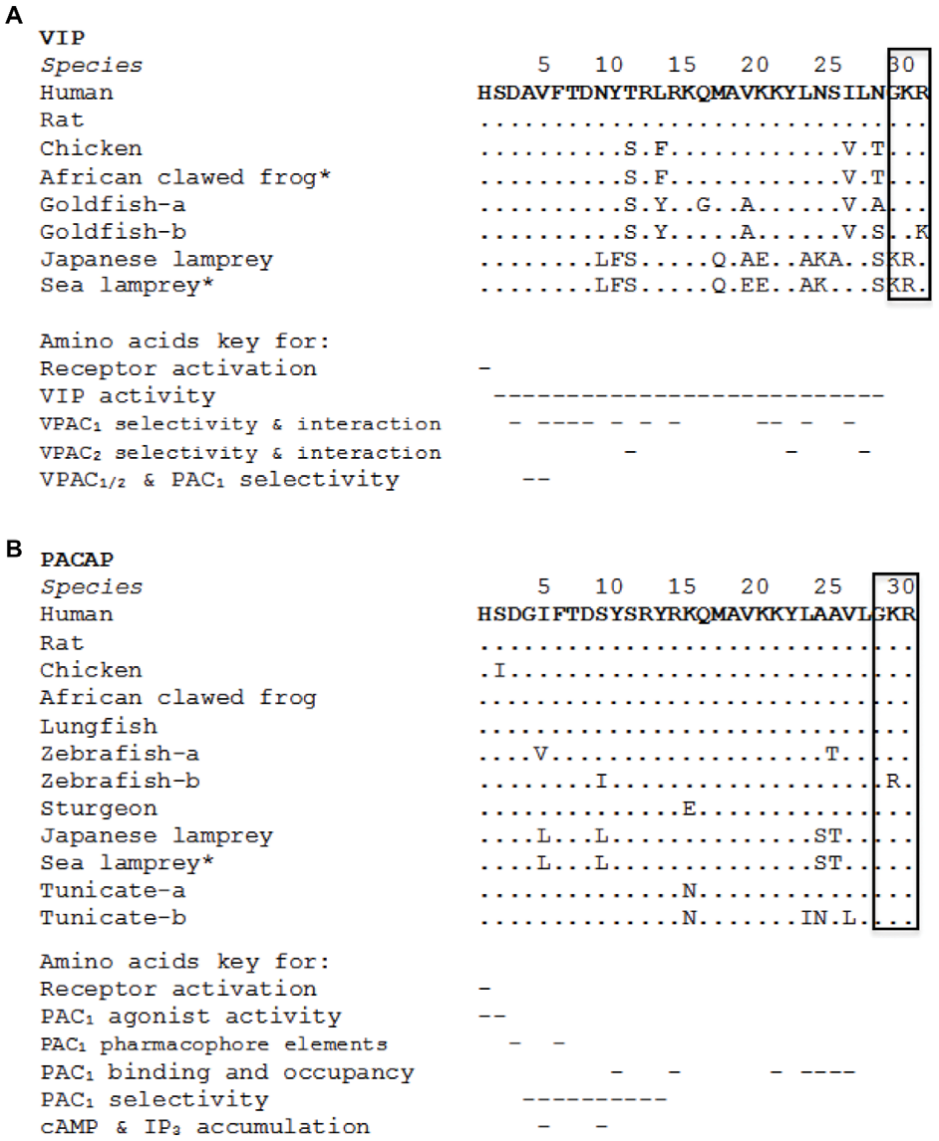
Amino acid alignment of VIP and PACAP-27 peptide sequences and C-terminal processing sites from various chordates. The amino acid alignment of (A) VIP and (B) PACAP-27 were generated using the default settings of Mega 5.0 software. Predicted sequences are denoted by “*” and retrieved from Ensembl or PreEnsembl online genome databases. The tribasic processing sites are boxed. Identical residues to that of human VIP and PACAP are denoted by “.”. Residues indicated important for selectivity and interaction based on mammalian studies are denoted by “–” [28], [44], [46], [61]–[72].

**Figure 2:**
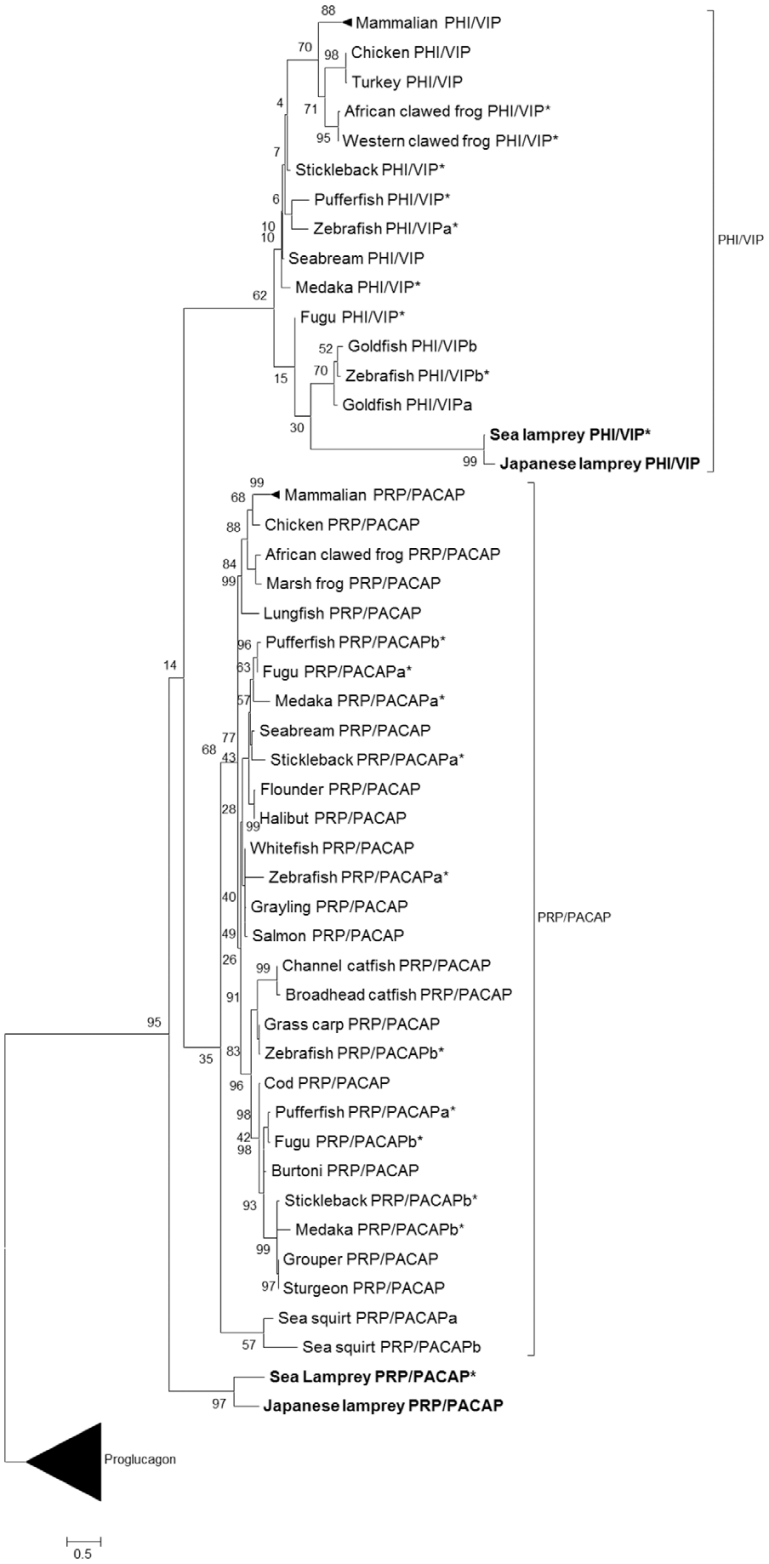
Phylogenetic analysis of PHI/VIP and PRP/PACAP hormone precursors. The tree was constructed by the PAM Matrix (Dayhoff) model by Maximum-Likelihood method, MEGA 5.0 software. Predicted sequences from the Ensembl genome database are denoted by “*”. The deduced sequences from this study are boldfaced. The numbers above each branch indicate the percentage of bootstrap replications in which that branch was found based on 500 replications. Proglucagon sequences were used as the outgroup.

By bioinformatic analyses, partial VIP and PACAP precursor cDNAs were predicted from the available sea lamprey preassembled genome. With the basis of typical secretory peptide cleavage sites (KRR/GKR) [25], a 28-amino acid VIP and a 27-amino acid PACAP were predicted. Alignment of the mature sea lamprey VIP and PACAP with other vertebrate sequences (Figure 1) showed high sequence homology with 57.1% and 66.7% identity respectively. Primers were designed according to these partial cDNA predictions to amplify VIP and PACAP cDNAs in the Japanese lamprey, obtaining two full length (jlpPHI/VIP, jlpPRP/PACAP) sequence(s) (Figure S3). For the Japanese lamprey, the full length putative PHI/VIP precursor (jlpPHI/VIP) cDNA was 1107 bp with an open reading frame of 504 bp encoding a 168-amino acid mature protein (inclusive of a 24-amino acid PHI and 28-amino acid VIP), whilst the full length putative PRP/PACAP precursor (jlpPRP/PACAP) was 760 bp in length with a 555 open reading frame encoding a 185-amino acid protein (inclusive of a 45-amino acid PRP and 27-amino acid PACAP). As mature protein sequences are too short to generate a reliable phylogenetic tree, precursor sequences of PHI/VIP and PRP/PACAP were used instead (Figure 2). In addition, proglucagonsequences were used as the outgroup. Phylogenetic analysis grouped the identified PHI/VIP and PRP/PACAP sequences from this study into each of their monophyletic groups accordingly. Structurally, the identified agnathan transcripts possess features typical of peptide hormones [26] and are homologous to their vertebrate counterparts (Figure S4).

### Ancestral VIP/PACAP receptors in *L.japonicum* and *E.burgeri*

**Figure 3:**
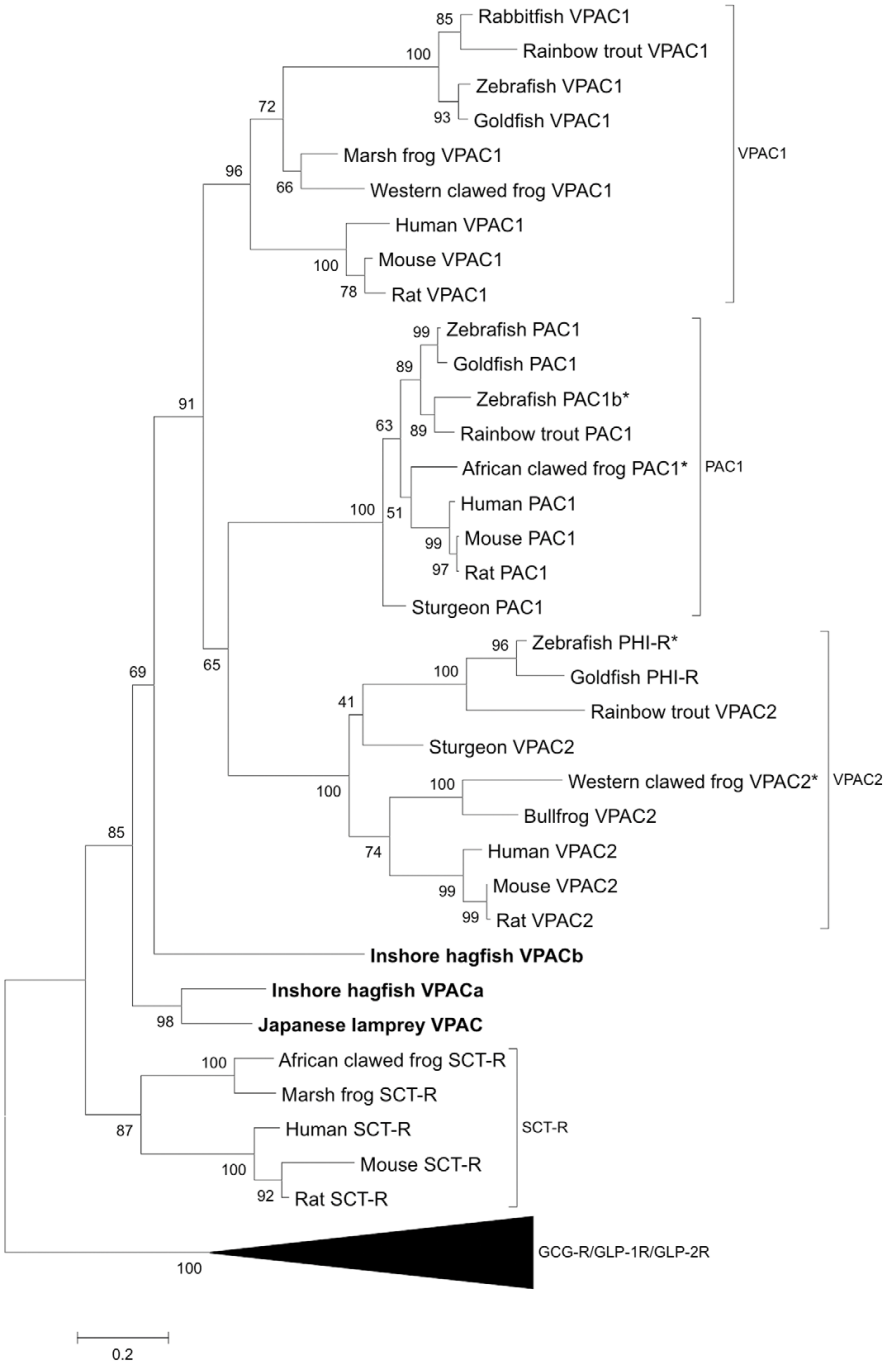
Phylogenetic analysis of vertebrate VIP/PACAP receptors (VPAC_1_, VPAC_2_ and PAC_1_). The tree was constructed based on the PAM Matrix (Dayhoff) model by Maximum-Likelihood method, MEGA 5.0 software. The monophyletic groups are indicated on the right. Cloned receptor sequences from this study are boldfaced. Predicted sequences from the Ensembl genome database are denoted by “*”. The numbers above each branch indicate the percentage of bootstrap replications in which that branch was found based on 500 replications. Glucagon, GLP-1, GLP-2 and GIP receptor sequences were used as the outgroup.

To amplify the VIP/PACAP receptors in *L.japonicum* and *E.burgeri*, degenerate primers were designed accordingly. The full-length putative *L.japonicum* VPAC receptor (jlpVPAC) cDNA obtained was 1860 bp with an open reading frame of 1440 bp encoding a 480-amino acid protein (Figure S5A). Two full-length putative *E.burgeri* VPAC receptor cDNAs were obtained (hfVPACa and hfVPACb) (Figure S5B and S5C): hfVPACa was 1847 bp with an open reading frame of 1404 bp encoding a 468-amino acid protein; and hfVPACb cDNA was 2190 bp with an open reading frame of 1359 bp encoding a 453-amino acid protein. Using the SignalP and TMHMM programs from CBS Prediction Server (http://www.cbs.dtu.dk/services/) (data not shown) and amino acid alignments (Figure S6), the putative receptors were shown to structurally resemble other class II B GPCRs possessing N-terminal signal peptides of 19- and 24-amino acid respectively for jlpVPAC and hfVPACs, followed by a ligand binding domain, seven transmembrane domains and a C-terminal cytoplasmic region. Phylogenetic analyses of the receptors were performed using the PAM matrix (Dayhoff) model by Maximum Likelihood method (Figure 3). PRP, GHRH and SCT receptors were included for their closer phylogenetic relationships with VIP/PACAP receptors, while glucagon (GCG), glucagon-like peptides (GLP-1 and GLP-2) and gastric inhibitory polypeptide (GIP) receptors were used as the outgroup. The tree grouped the putative jlpVPAC and hfVPACs to the clade of VIP/PACAP receptors, supporting their identities as the orthologues of non-agnathan forms and the overall scenario agrees with a previously proposed scheme [27]. As the Agnathan receptors did not group individually into either of the VPAC_1_, VPAC_2_ or PAC_1_ subgroups, all three VIP/PACAP receptor subtypes (inclusive of a range of vertebrate species) were used as a comparison (Figure S7). Amongst the agnathan receptors, jlpVPAC and hfVPACa are more similar being grouped together, sharing 65.6% homology and a range of common structural motifs (Figures 3, S6, and S7). Based on human VPAC_1_ studies, the highly conserved residues Glu^36^, Asp^48^, Trp^−73^, Pro^87^, Trp^110^ and Pro^115^ essential for VIP binding [28]–[31]; and putative salt bridge between Asp^68^ and Arg^103^ important for receptor fold stabilization and essential for ligand binding [30] are conserved in both jlpVPAC and hfVPACa. In addition, the consensus SE-R/K motif for phosphorylation [32], YL amino acid pair for G-protein coupling [33], RL-A/V-K motif for G_αs_ coupling [34], [35], [36], IIRIL motif unique only to VIP binding receptors and PD-I/V motif for VIP binding are also present in jlpVPAC and hfVPACa. Though hfVPACb shares majority of the conserved structural features mentioned, some residues are either conservatively or drastically substituted. These include alteration of residues described necessary in human VPAC_1_ VIP binding (36: Glu → Asp and 87: Pro → Trp), however the similar chemical nature of the substitutes may not affect hfVPAb's binding ability of VIP drastically. In contrast, some motifs including SE-R/K and PD-I/V are partially present, which together may have a combined effect on the receptor's signal transduction mechanisms. This may account for hfVPACb's contrast in signalling abilities to hfVPACa and jlpVPAC, it was found unable to stimulate adenylate cyclase accumulation but able to elicit the calcium pathway as shown in the later part of this manuscript.

### Differing signalling capabilities between the hfVPAC receptors

**Figure 4:**
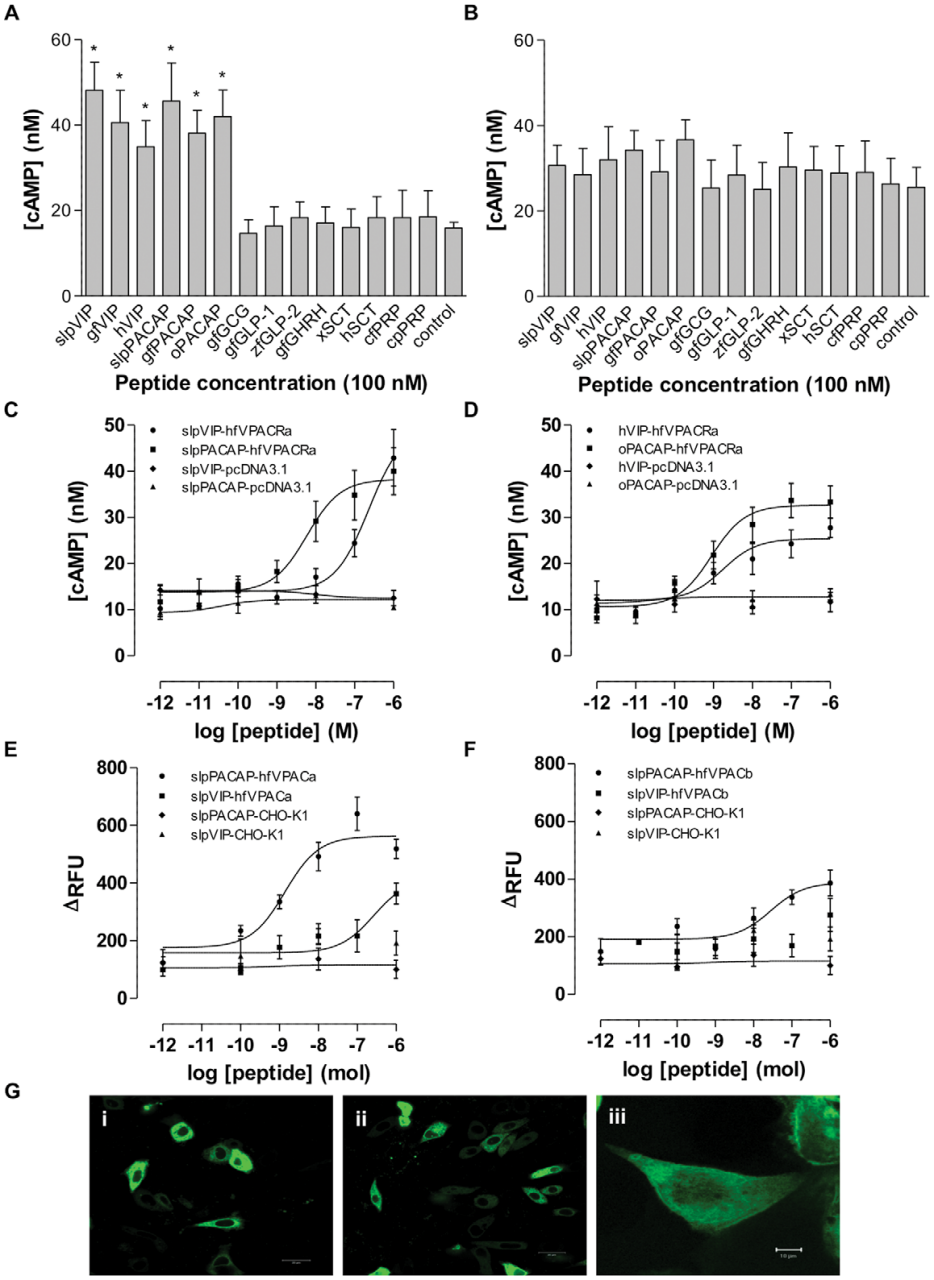
Functional characterization of hfVPACa and hfVPACb. Intracellular cAMP accumulation in response to 100 nM vertebrate superfamily peptides on COS-7 cells transiently transfected with (A) hfVPACa and (B) hfVPACb. Peptide species: slp, sea lamprey; gf, goldfish; h, human; o, ovine; zf, zebrafish; x, *Xenopus*; ct, catfish and cp, carp. Data represent the mean ± S.E.M. of at least 4 experiments performed in duplicates, p<0.01 is denoted by “*”. Effects of graded concentrations of (C) agnathan and (D) mammalian VIP and PACAP peptides on COS-7 cells transiently expressing hfVPACa. Data are expressed as the mean ± S.E.M. of at least 6 experiments performed in duplicates. Measurement of intracellular calcium elevation in CHO-K1 cells transiently expressing (E) hfVPACa and (F) hfVPACb in response to graded concentrations of sea lamprey PACAP. Data are expressed as the mean ± S.E.M. of at least 4 experiments. RFU, relative fluorescence units. (G) Shown are representative of confocal fluorescence images of CHO-K1 cells expressing (i) hfVPACa-pEYFP, (ii) hfVPACb-pEYFP and (iii) pEYFP-N1.

To investigate the functional properties of the proteins encoded by the agnathan VPAC receptor cDNAs, various vertebrate secretin superfamily peptides at a 100 nM concentration were employed to determine their abilities to stimulate cAMP production in transiently transfected COS-7 cells with hfVPACa or hfVPACb. As jlpVPAC was indicated to be orthologous to hfVPACa by phylogenetic analysis, only the hfVPAC receptors were tested (Figure 4). All VIP/PACAP peptides applied have the ability to stimulate and accumulate cAMP in cells expressing hfVPACa, whilst other peptides such as glucagon, GLP-1, GLP-2, GHRH and SCT were weakly or unable to do so (Figure 4A). In contrast, none of these peptides were able to significantly stimulate cAMP production in hfVPACb transfected cells (Figure 4B). The hfVPACa was further studied using graded concentrations of VIP/PACAP peptides from various vertebrates including sea lamprey (Figure 4C and 4D). The half maximal effective concentrations (EC_50_) are 0.895 nM ovine PACAP-27 >1.74 nM human VIP >>5.92 nM sea lamprey PACAP-27.

Aside from the adenylyl cyclase pathway, VIP/PACAP receptors particularly the PAC_1_ receptor are known able to stimulate calcium mobilization either directly or via the PLC pathway [9], [37]. Together with data from the cAMP assay showing hfVPACb not being responsive in adenylyl cyclase accumulation, a second assay involving calcium (Fluo-4 calcium assay) was therefore considered (Figure 4E and 4F). Cells expressing the hagfish receptors showed a remarkable increase in intracellular calcium in contrast to the controls (no peptide stimulation or vector-transfected cells). However, hfVPACb is less sensitive to peptide stimulation and has an EC_50_ of 27.8 nM compared to hfVPACa (1.39 nM) (in response to sea lamprey PACAP). To determine if the differences in signal transduction ability between the hfVPAC receptors was due to variations in expression or problems in receptor trafficking in culture cells, confocal microscopy studies were performed. Both receptors and the positive control (pEYFP-N1), but not the pcDNA3.1 vector transfected control (data not shown) were able to traffic to the cell surface for expression, resulting in fluorescent signals on the cell surface of CHO-K1 cells (Figure 4G).

### hfVPAC receptors are localized predominantly in the brain

**Figure 5:**
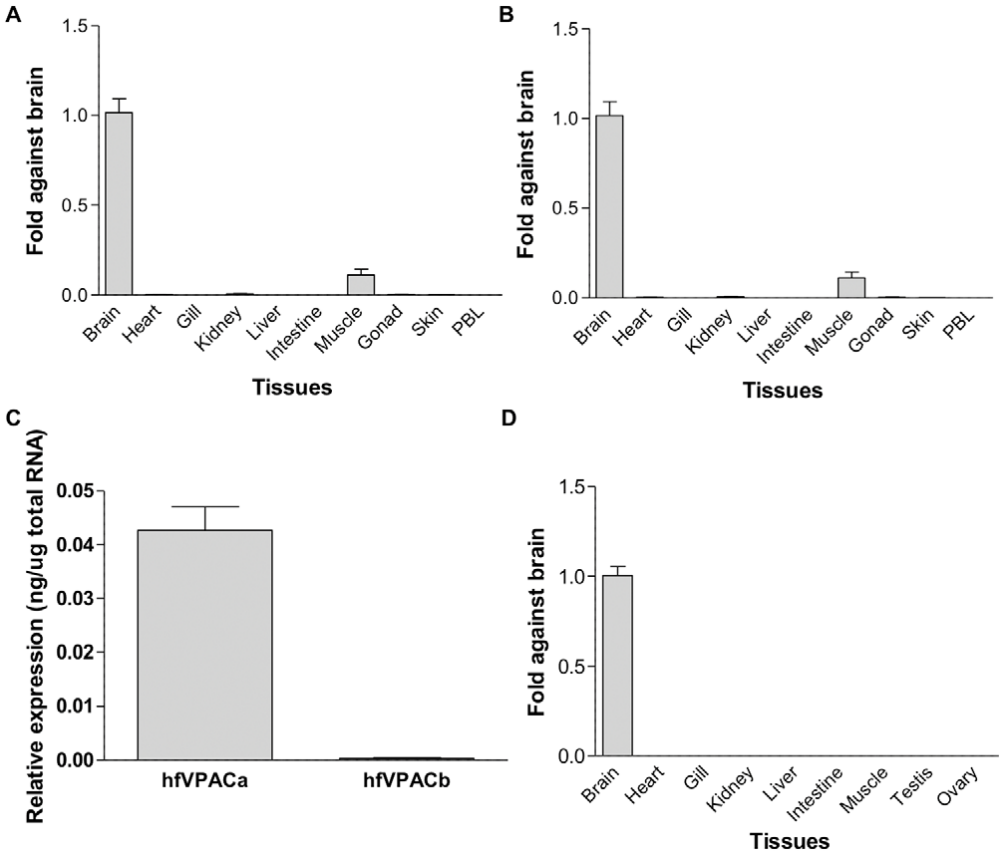
Transcript expression profile of agnathan VPAC receptors and PACAP. Tissue distribution patterns of (A) hfVPACa and (B) hfVPACb; a relative abundance of 1 was set arbitrarily for the mRNA expressed in the brain. (C) The expression level of hfVPAC receptors in the hagfish brain was calculated from respective standard curves. (D) Tissue distribution pattern of jlpPACAP; a relative abundance of 1 was set arbitrarily for the mRNA expressed in the brain. Data are expressed as the mean ± S.E.M. of four experiments performed in duplicates.

Regarding the expression profiles of hfVPAC receptor transcripts in various tissues, real-time PCR revealed strongest expressions of both hfVPACa and hfVPACb transcripts in the hagfish brain (Figure 5A and 5B). Within the brain, hfVPACa has a higher expression level of 42.6±4.4 pg/µg total RNA compared to hfVPACb (0.4±0.2 pg/µg total RNA) (Figure 5C). In other tissues, hfVPACa transcripts could be observed in the muscle and kidney, but was absent from the heart, gill, liver, intestine, gonad, skin and peripheral blood leukocytes (PBL). hfVPACb transcripts were found only weakly in the kidney, intestine, muscle, and gonad, and were absent in the heart, gill, liver, skin and PBL (Figure 5B).

**Figure 6:**
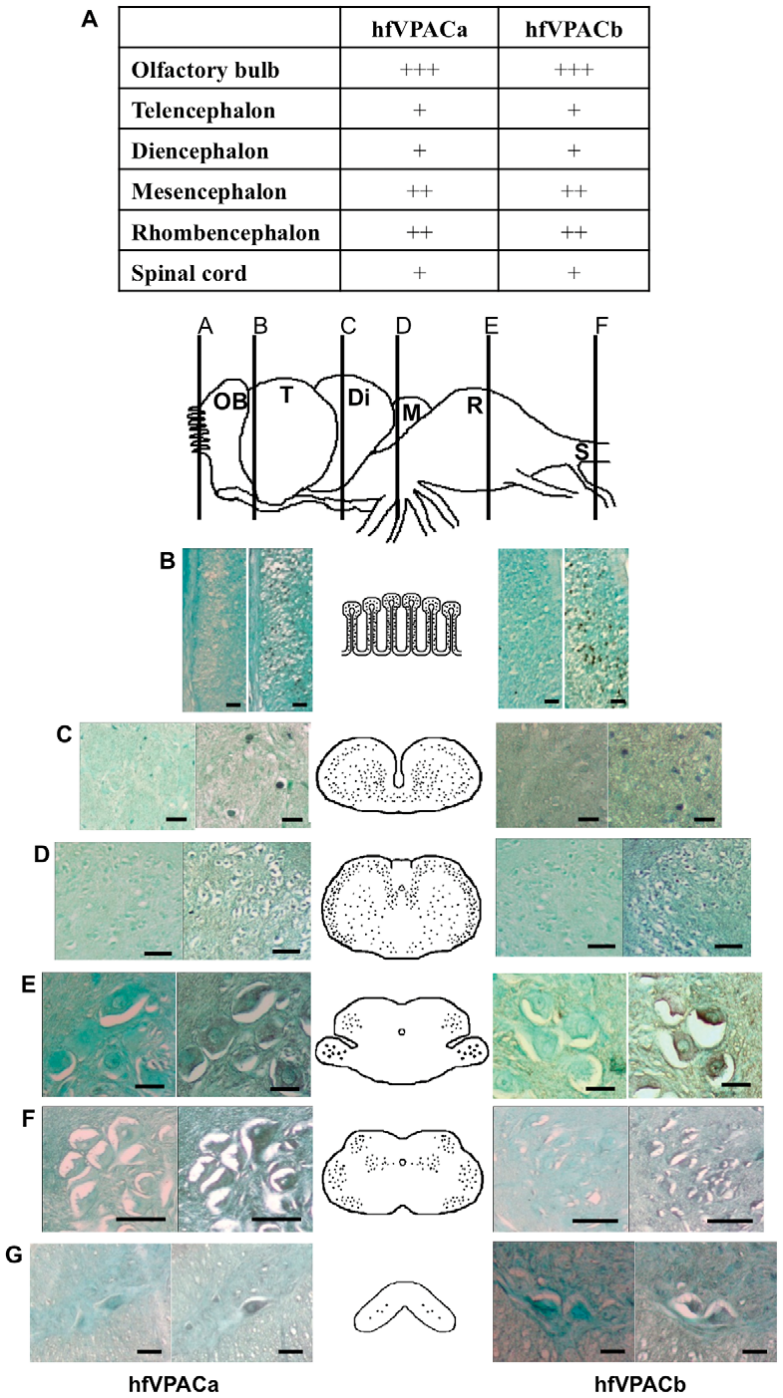
*In situ* hybridization analysis of hfVPAC mRNAs in the brain of *E.burgeri*. (A) Summary of relative abundance of hfVPACa and hfVPACb receptor expressions in various brain regions: High (+++), moderate (++), low (+), very low (–).The images and schematic diagrams show the distribution of hagfish mRNA in collateral sections of several brain regions (B–G). From left to right of the schematic diagrams, hfVPACa and hfVPACb images inclusive of their negative controls using a 1∶30 ratio of DIG-labeled anti-sense probe and unlabeled anti-sense probe and positive signals using specific complementary probes are shown. Fast green was used for counterstaining. Scale bars, 0.25mm for B and D; 0.15mm for C, E–G.

With real-time PCR indicating the brain to have highest expression of hfVPACa and hVPACb, *in situ* hybridization histochemistry was performed in the hagfish brain to reveal the cellular distribution of these receptors. Positive hybridization signals were observed throughout the hagfish brain, showing the widespread distributions of hfVPACa and hfVPACb transcripts (Figure 6A). For both receptors, strong hybridization signals were most abundantly observed in the olfactory bulb (OB) region (Figure 6B), extensively in the small circular mitral cells, which are the predominant OB cell type. In the telencephalon (Figure 6C), the hagfish transcripts were dispersed throughout the statrum griseum superficiale (pars compacta, pars lateralis and pars parvocellularis) and central prosencephalic complex. Positive signals for both receptor transcripts were also found moderately dispersed in small nuclei cells in the diencephalon (Figure 6D). In the mesencephalon and rhombencephalon (Figure 6E and 6F), positive signals were found predominantly in larger nuclei cells such as in the nucleus radicis motorius nervi facialis and nucleus motorius magnocellularis nervi trigemini. Low levels of signals were also observed throughout the spinal cord in large teardrop shaped nuclei (Figure 6G).

## Discussion

### Newly identified agnathan ligands are ancestral forms to vertebrate VIP/PACAPs

Previously, two PACAP cDNAs were identified in the tunicate and considered as the most ancient PACAPs to be reported [16]. It was suggested that a more recent gene duplication event was responsible for the origin of the two tunicate PACAP genes and the ancestral gene of these two genes was the precursor to all vertebrate PRP/PACAP genes [16]. The authenticity of these PACAP sequences was however questioned [17], [18], [19] due to the fact that neither PACAP nor VIP/PACAP receptor sequences were found in any of the invertebrate genomes or EST databases. Having discovered an assortment of agnathan PHI/VIP and PRP/PACAP sequences, we attempted to fit our findings into the preexisting hypothesis so as to bridge the transition from protochordates to gnathostomes. Phylogenetic analysis grouped the agnathan PHI/VIP and PRP/PACAP ligands into their monophyletic clusters, suggesting specific ligand forms had already evolved and highly likely associated with the VIP/PACAP ligand family. Sequence alignments ranging from tunicate to mammalian species showed typical organizations of mature peptide sequences (Figure S4) [26] and conservation of majority of residues described to be important (Figure 1), thus reaffirming VIP and PACAP's functional importance in early vertebrates. The agnathan PACAPs are comparable to the tunicate forms, existing only as 27-amino acid peptides which are preceded immediately by “GKR” sites. Taken together with the variability of the sequences beyond these first 27 amino acids and the lack of further processing sites, it seems that an extended PACAP-38 form is unlikely in agnathans. As the first 27 amino acids are found necessary for carrying out PACAP's biological activity [38], this suggests that the ancestral PACAP emerged as a 27-amino acid form before being elongated later in vertebrate evolution to possess two processing sites, allowing for processing of both PACAP-27 and PACAP-38. It is in the Chondrichthyes fish class which PACAP-38 may have emerged, as supported by purification of PACAP which has “GKR” and “GRR” processing sites for processing of both ligand forms in the stingray (*Dasyatis akajei*) [39].

Being the closest extant outgroup to all jawed vertebrates, we envisaged the agnathan PACAP precursors to be structurally intermediate to the tunicate-protochordate and vertebrate forms. Nevertheless, our phylogenetic tree showed our agnathan PACAP positioned in the initial branch leading to the PHI/VIP and PRP/PACAP precursor groups prior to the tunicates (Figure 2), suggesting that the agnathan PACAP precursor as phylogenetically more ancient. With such puzzling findings, we questioned if our analysis was biased and attempted to resolve this by considering various parameters when constructing our phylogeny, however the analyses obtained showed either a similar scenario or evolutionary patterns unfavorable to existing hypotheses [27], [40]–[43] with regard to VIP/PACAP evolution and genome duplication (data not shown). Aside from phylogenetic data, similar to Cardoso's group [19], searches using sequences from the vertebrate secretin superfamily members and the duplicate tunicate PACAPs failed to identify sequences or structural homologues in the genomes or EST databases of porcifera, cnidaria, protostome and early deuterostomes [19]. As an alternative, a secretin superfamily prototype sequence model was also used by Cardoso's group to search against invertebrate and vertebrate genomes and EST sequences, however, the only homologues found were from vertebrates [19]. Taking these evidences together, the authenticity of previously reported invertebrate PACAP sequences remains unconfirmed, while we can confirm here that the newly identified agnathan VIP and PACAP genes are ancestral to all known vertebrate forms.

### Evolution of the VIP/PACAP genes

**Figure 7:**
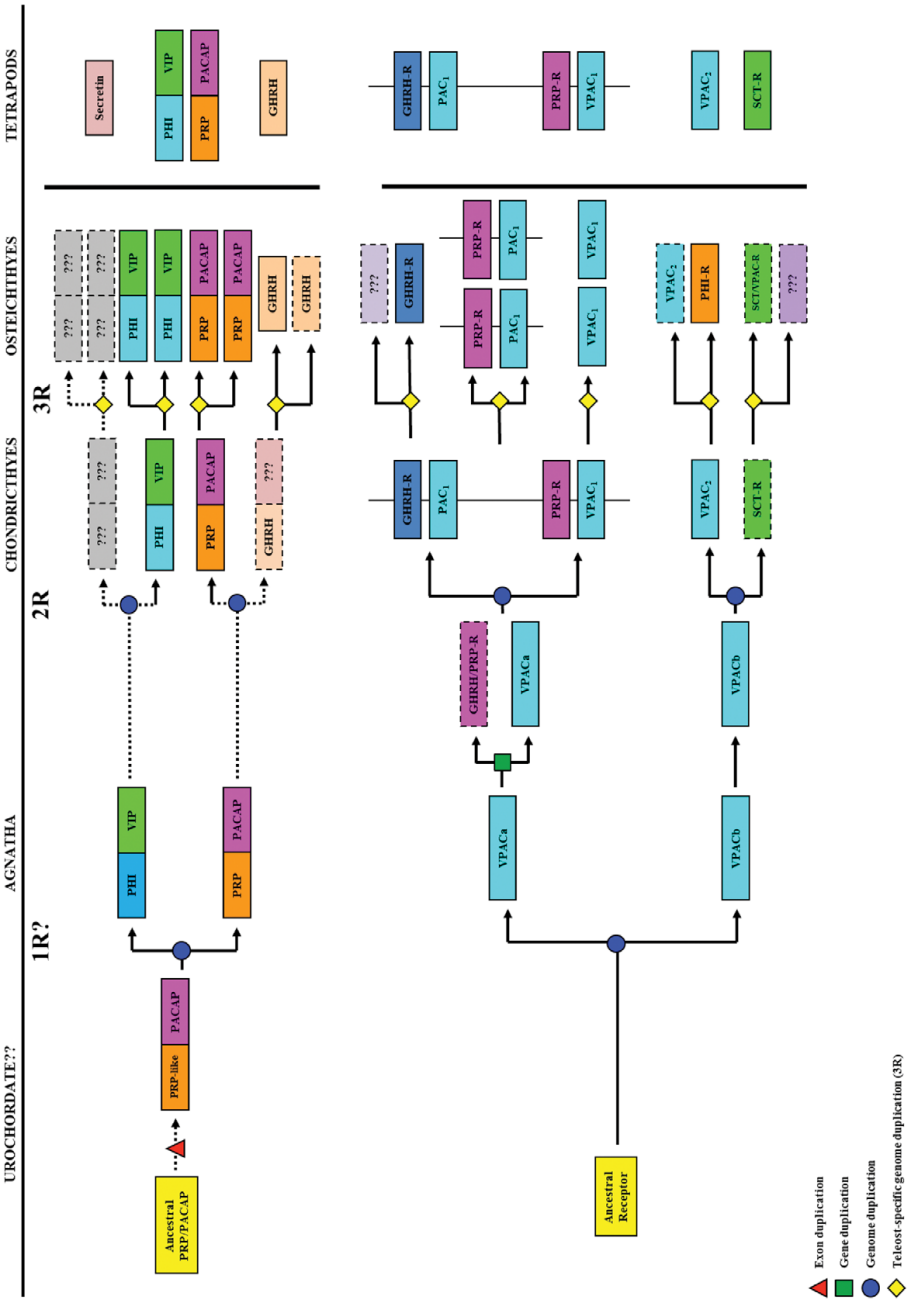
An evolutionary scheme of the VIP/PACAP ligands and receptors in vertebrates. The boxes denote exons for the ligands and genes for the receptors. Unknown or unclear events are denoted by dotted lines or question marks. The phylogenetic timeline for the events are not to scale.

Synonymous with other vertebrate VIP/PACAP, the agnathan peptides are under strong evolutionary pressure, maintaining well-preserved loci of biological activity in their N-terminal domains [44], [45], [46]. On the basis of sequence similarities and chromosomal organizations, genes encoding these peptides are proposed to have a common evolutionary origin, arising from an ancestral gene more than 650 mya before exon duplication, gene and/or genome duplications to generate the present forms [27], [41], [42], [43]. Ohno's whole genome duplication model although not the timing of duplications is a widely accepted model to explain the evolution of many vertebrate genes. Based on more recent studies, a primordial gene is likely to have undergone two rounds of genome duplication approximately 500 to 800 mya [15], giving rise to four paralogous genes (PHI/VIP, PRP/PACAP, GHRH and SCT) [19], [27], [47] within which the first early vertebrate PHI/VIP and PRP/PACAP forms are likely those identified in this study (Figure 7). These findings also suggest that VIP and PACAP are physiologically important even in early vertebrates, although their structures were only more stringently conserved later in vertebrate evolution as demonstrated by the high levels of sequence identity shared amongst non-agnathan vertebrate VIP and PACAP.

### Agnathans possess ancestral VIP/PACAP receptor subtypes

Likewise, the VIP/PACAP receptors from the agnathan fish models studied sheds some light on the early evolutionary events leading to the occurrence of vertebrate VPAC1, VPAC2 and PAC1 receptors. From our study, the cloned receptors not only possess emblematic GPCR characteristics, but are also shown to be phylogenetically most ancient, being located on the initial branches of the VIP/PACAP receptor group, but are unable to group distinctly into either of the atypical VIP/PACAP receptor subgroups (VPAC1, VPAC2, PAC1) in the phylogenetic tree (Figure 3). Of the two hagfish receptors, hfVPACb is grouped separately from jlpVPAC and hfVPACa. Functionally, hfVPACa showed a clear preference towards VIP and PACAP in cAMP stimulation (Figure 4A), generally exhibiting lower EC50 values for PACAP than VIP (Figure 4C and 4D) (e.g. 0.895 nM ovine PACAP >1.74 nM human VIP) and is similar to human PAC1 pharmacological characteristics [48]–[51]. Of the PACAPs tested, the ovine peptide was found more effective than sea lamprey (5.92 nM) on hfVPACa, and may be accounted for by the conservation of key residues indicated to be important for binding, occupany and selectivity based on human PAC1 studies [50], [52], [53]. Taking together hfVPACa's ability to couple to both adenylyl cyclase and PLC pathways, it is likely a biologically functional VPAC receptor in the hagfish. On the other hand, hfVPACb was only able to transduce through the calcium pathway dose-dependently (Figure 4F), the physiological relevance of this remains to be investigated in the future.

### Evolution of the VIP/PACAP receptors

Previously, searches in invertebrate genomes and EST databases failed to identify sequence homologues with secretin superfamily receptors [17], [19], [54], [55], [56], suggesting that the superfamily of secretin receptors were evolved only during the rise of vertebrates. In this study, by blast searches of the lamprey and hagfish VPAC receptors against the amphioxus (*Branchiostoma floridae*) genome (data not shown), we have identified fragmentary sequences resembling several transmembrane domains of VPAC/PTH-like receptors. Our data therefore show the presence of VPAC/PTH-like receptor genes in invertebrates (Figure 7). Furthermore, recent completion of the sea lamprey genome has allowed us to utilize the identified jlpVPAC sequence to search against the sea lamprey database, revealing a total of 15 scaffolds with similar features (Table 1). With exception of scaffold GL501248, all others are found to share homology to GPCRs in the secretin receptor superfamily including glucagon, glucagon-like peptides and PTH receptors. This is useful in confirming the presence of receptors such as those from the secretin receptor superfamily even at an early stage in vertebrate evolution. Within the scaffolds identified, only GL478425, GL490074 and GL491710 share highest homology to the agnathan receptors identified and are useful in deciphering existence of VIP/PACAP receptor subtypes. GL478425 resembles the N-terminal, transmembranes 6 and 7 in both jlpVPAC and hfVPACa, amongst which jlpVPAC shares 100% identity. Taken with phylogenetic analysis grouping of these receptors together, this confirms the sea lamprey receptor in GL479425 as the orthologue of jlpVPACa. In contrast, GL490074 resembles transmembranes 6 and 7 of hfVPACb only and could therefore be the second subtype VPACb in lamprey. This was further supported by the low homology (37%) between GL490074 and GL478425, indicating that they belong to different genes. Lastly although GL491710 resembles transmembranes 3 and 4 of both jlpVPAC and hfVPACb, this fragment cannot be overlapped with either of the previously described scaffolds as they represent other transmembrane regions. It is therefore likely that GL491710 corresponds to a fragment of lamprey VPACb since it does not bear strong homology to jlpVPAC nor resemble hfVPACa. Alternatively, it may also be possible that GL491710 may represent part of a third VPAC receptor in the agnathan lineage. However, this seems unlikely as only two receptor subtypes have so far been identified in the hagfish and fit into the two whole genome duplications. Taking into account the “1–2–4” gene duplication rule, two of these genes may be represented by the two agnathan receptor subtypes identified in this study (Figure 7). With a second round of genome duplication, the agnathan VPAC receptors probably evolved into specific VPAC1, PAC1, VPAC2 receptor subtypes. The fact that both hagfish VPAC receptors are able to increase calcium levels may suggest that the ability for early VPAC receptors to mobilize and subsequently regulate calcium is essential. This also coincides with the fact that protostome putative GPCRs are found more similar to vertebrate receptors for calcitonin, calcitonin gene related peptide and corticotrophin releasing factor which are associated with calcium homeostasis and stress response [17].

**Table 1:** Sea lamprey scaffold search results.

Scaffold	Genscan result	Highest homology	Regions shared
GL478425	N.A.	jlpVPAC/hfVPACa	NT, TM6, TM7
GL491710	ENSPMAP00000000173	hfVPACb/jlpVPAC	TM3–TM4
GL476704	ENSPMAP00000009009	GluR	TM3–TM7
GL484465	ENSPMAP00000004226	GLP2R	NT–TM5
GL480593	ENSPMAP00000001499	PTHR2	TM3–CT
GL476336	ENSPMAP00000003315	PTHR1	NT–TM7
GL490074	N.A.	hfVPACb	TM6, TM7
GL480239	ENSPMAP00000003884	GLP1R	NT–TM2
GL476735	ENSPMAP00000000541	CALCRL	NT–CT
GL489095	ENSPMAP00000010121	GPR116	NT–TM7
GL477206	ENSPMAP00000009915	CRF2R	NT–CT
GL477106	ENSPMAP00000005701	GPR113	TM3–CT
GL476767	ENSPMAP00000004800	CRF2R	NT–CT
GL501248	ENSPMAP00000000521	TRP–16	Not GPCR
GL477374	ENSPMAP00000009497	Latrophilin 2-like	NT–CT

It has been hypothesized that molecular and evolutionary diversity are interlinked and this is well delineated in the secretin receptor superfamily which are the largest and most versatile assortment of transmembrane receptors in the cell [12]. Duplication events whether occurring at the exon, gene or even genome level are necessary driving forces in creating sources of novelty and as a result allowing new functions to arise and evolve. Often the functions that are elicited are a result of ligand-receptor pair interactions; therefore it is important not only to study the evolution of ligands and receptors independently but also to examine their relationships together. In this study, the first VIP/PACAP ligands and receptors have been identified in the early vertebrate jawless fish models: lamprey and hagfish. Together with evidence from invertebrate genomes and EST sequences, it can be hypothesized that the VIP/PACAP receptors may have more ancient origin than the ligands [19]. Gene or genome duplications likely gave rise to the duplicate VIP/PACAP receptors in the agnathans that are able to interact with VIP and PACAP. Of the two duplicates, hfVPACa was likely more predominant in terms of function as demonstrated by its higher abundance in the brain (∼100 fold total RNA) (Figure 5C). With molecular diversity correlated to organismal complexity, the initial functions of both VIP and PACAP are likely to originate in the brain as a neuropeptide as supported by our data showing the brain to predominate in lamprey PACAP expression (Figure 5D), before evolving to become pleiotropic peptide hormones with widespread distribution throughout the body later in vertebrate evolution. This is likely complemented by development of the intricate central nervous system, whereby agnathans lack hypophysiotropic neuron innervation of the pituitary and a median eminence capillary network as a means to communicate between the brain and pituitary, of which they are found in teleost fish and some bony fish/amphibians/reptiles/birds/mammals respectively [40], [57]. Taken together with our findings, VIP/PACAP are likely key in eliciting central roles, whilst having peripheral effects via release into the circulation or simple diffusion.

In agnathans, the presence for two PACAP forms (PACAP-27 and PACAP-38) had not yet evolved. Despite this, VIP and PACAP are under strong selective pressure indicated by the highly conserved structures of their mature peptides, highlighting the importance of their functions early in evolution. It is the occurrence of successive genome/gene duplication events which drove the evolution of the duplicate VPAC receptors into more specific receptor subtypes, with one of the VPAC duplicates later becoming VPAC_1_ and PAC_1_, whilst the other becomes VPAC_2_ [14], [58]. This also indicates that more specific receptor subtypes are probably required to elicit functions at a more complex level, particularly since the progression towards other vertebrates such as mammals is associated with an increased need for regulation of physiological processes. The status of the invertebrate members of the secretin superfamily remains uncertain, although the present report of VPAC/PTH-like receptor in an invertebrate suggests further research on functional aspects of the ligands and receptors is needed. Overall, the VIP/PACAP ligands and receptors have evolved via tandem duplication events, becoming more stringent in structural conservation and specialization to accommodate to the enhancement of vertebrate intricacy during evolution.

## Conclusions

The molecular evolution of ligand-receptor pairs is an interesting aspect where much effort has been put forth in attempts to unravel these evolutionary episodes. However, determining these earlier events is often complicated by secondary losses of genes or chromosomes, chromosomal rearrangements, independent gene duplications and differences in evolutionary rates for various genes and the lack of knowledge particularly in early vertebrate forms poses another obstacle. To investigate these enigmatic events, this study reports the first agnathan VIP/PACAP ligand-receptor pairs. Their structures have been closely dissected and their functional capabilities examined. The lamprey VIP/PACAP ligands are shown to have high conservation of their mature peptide sequences, further highlighting the strong evolutionary pressures, which persist throughout vertebrate evolution to preserve structure and ultimately function. The cloning of their receptors however provide some newer information, illustrating structures which are likely closer representatives of the ancestral forms possessing mixed features from the well-defined VPAC_1_, VPAC_2_ and PAC_1_ subtypes. It is interesting to discover that one of the hagfish VPAC receptors cannot signal transduce via the cAMP pathway but able to increase intracellular calcium levels. Furthermore, high abundance of VIP/PACAP receptors identified in the hagfish brain suggests that the first functions of VIP and PACAP are most likely targeted to the brain. With the enhancement of physiological systems in vertebrate evolution, VIP/PACAP ligands and receptors evolved accordingly resulting in even higher sequence constraints necessary for specific receptor subtypes to carry out their pleiotropic functions.

## Methods and Materials

### RNA samples and first strand cDNA

Adult Japanese lampreys (*Lampetra japonicum*) and inshore hagfish (*Eptatretus burgeri*) were purchased from Ebetsu's Fisherman's Union and Sekikatsu Company, Hokkaido, Japan respectively. Total RNA of Japanese lamprey (*Lampetra japonicum*) and inshore hagfish (*Eptatretus burgeri*) were isolated by Trizol reagent (Invitrogen, Carlsbad, CA). First strand cDNA was synthesized from 5 μg total brain RNA using SuperScript^TM^ III RT (Invitrogen). Rapid amplification of cDNA ends (RACE) was performed using the 5′ and 3′ RACE (Invitrogen); and GeneRacer^TM^ (Invitrogen) kits respectively for lamprey and hagfish. Four inshore hagfish were also captured in the Pacific Ocean off the coast of Miura Peninsula, Kanagawa, Japan from which the brains were fixed in formalin for *in situ* hybridization studies.

### Molecular cloning of VIP and PACAP from *L.japonicum* and VPAC receptors from lamprey and hagfish *E.burgeri*

Degenerate primers for the amplification of Japanese lamprey VIP and PACAP (jlpVIP, jlpPACAP), Japanese lamprey VIP/PACAP receptor (jlpVPAC) and hagfish VIP/PACAP receptor (hfVPAC) were designed according to conserved regions of aligned VIP, PACAP and VIP/PACAP receptor sequences obtained from the Ensembl and NCBI databases (Table S1). RACE was performed using specific primers designed according to the partial sequences. Full-length cDNA clones encompassing the 5′ to 3′ untranslated regions were produced by PCR with specific primers and confirmed by DNA sequencing. Full-length hfVPAC cDNA was subcloned to pcDNA3.1+ (Invitrogen) for functional expression.

### Tissue Distribution of PACAP in *L.japonicum* and VIP/PACAP receptors in *E.burgeri*

Quantitative real-time PCR was used to determine the expression profile of PACAP and VPAC receptors in various tissues of *L.japonicum* and *E.burgeri* respectively. After synthesis of first-strand cDNAs, RT-PCR (n = 4, each in duplicates) was performed using the Power SYBR Green PCR Master Mix (Applied Biosystems, Foster City, CA) and the 7300 Real Time PCR System (Applied Biosystems). Primers used in the real-time PCR are listed in Table S1. The threshold cycle (Ct) is defined as the fractional cycle number at which the fluorescence reaches 10-fold standard deviation of the baseline (from cycle 3 to 10). The specificity of the SYBR PCR signal was confirmed by both melt curve analysis and agarose gel electrophoresis. Standard curves for the VPAC receptors were established by 10x serial dilution of hfVPAC-pcDNA3.1+ plasmid stocks.

### Peptides

Glucagon (goldfish), glucagon-like peptides (goldfish and zebrafish), GHRH (goldfish), PRP (catfish and carp), PACAP (goldfish and ovine) peptides were synthesized by the Laboratory of Cellular Physiology and Immunology, Rockefeller University (New York, NY). VIP peptides were synthesized by Bachem California (Bachem California, Inc., CA). Human SCT was bought from AnaSpec (AnaSpec, Inc., CA). *Xenopus* SCT and sea lamprey VIP and PACAP (predicted amino acid sequences from *P.marinus* preassembled genome) peptides were synthesized by Alain Fournier in collaboration with Prof. Hubert Vaudry from the Institut National de la Santé et de la Recherche Médicale U413, European Institute for Peptide Research, University of Rouen (Rouen, France). All synthetic peptides are of >95.0% purity.

### Transient expression of hfVPAC receptors in COS-7 and CHO-K1 cells

African green monkey kidney (COS-7) and Chinese hamster ovary (CHO-K1) cells (ATCC, Manassas, VA) were cultured in 10% FBS/100 U/ml Penicillin/100 g/ml Streptomycin supplemented DMEM and MEM respectively in a 5% (v/v) CO_2_ humidified chamber at 37°C and passaged twice per week using Trypsin-EDTA (TE) (Invitrogen) on Nunc^TM^ Surface (Nunc, Denmark) tissue culture flasks. For cAMP and calcium studies, COS-7 or CHO-K1 cells were seeded at a density of 2.5×10^5^ cells/well in 6-well plates 48 hours prior transfection. 4 µg hfVPACa-pcDNA3.1 and hfVPACb-pcDNA3.1 expression constructs were transfected into cells using 12 µl GeneJuice Transfection Reagent (Novagen, Darmstadt). Control cell lines were established by transient transfection with pcDNA 3.1 (+) vector (Invitrogen). For cAMP assays, COS-7 cells were stimulated for 30 mins with either fixed (10^−6^ M) or varying (10^−6^ to 10^−12^ M) concentrations before taking measurements using the LANCE cAMP assay kit (Perkin-Elmer, Waltham, MA) in the Victor x4 multilabel reader (Perkin-Elmer) according to the manufacturer's protocol. Intracellular cAMP levels ([cAMP_i_]) were measured and expressed as cAMP concentration relative to the basal level (stimulation buffer alone without peptide addition). Negative control experiments were performed using control COS-7 cell line in each experimental trial.

For calcium assay, transiently transfected CHO-K1 cells were rinsed with MEM/10% FBS/100 U/ml Penicillin/100 g/ml Streptomycin and lifted with TE 48 hours after transfection. Cells were then reseeded at a density of 1 x 10^5^ cells/well in black 96-well plates overnight. Prior to performing calcium assay, cells were rinsed twice with prewarmed solution 1 (2.5 mM Probenecid (Invitrogen) dissolved in 250 mM NaOH and HBSS (Invitrogen)) before incubation with solution 2 (0.5% Fluo-4 and 0.1% pluronic solution (Invitrogen) in solution 1) at 37°C for 1 hour. The cells were then rinsed and supplemented with solution 1. Peptides varying in concentrations from 10^−6^ to 10^−12^ M were added and calcium levels were traced in a real-time manner using the Victor x4 multilabel reader at 37°C. Data were expressed in ΔRFU value (maximum changes in fluorescence signals from baseline). Negative control experiments were performed using control CHO-K1 cell line in each experimental trial.

For confocal fluorescent imaging studies, CHO-K1 cells were seeded at a density of 1.0×10^5^ cells/well on 12-mm poly-D lysine coated coverslips 48 hours before transfection using conditions previously described. Transiently transfected cells were rinsed with ice cold HBSS and fixed with paraformaldehyde (PFA). Coverslips were then lifted and mounted onto glass slides with 1 × Tris-buffered saline and secured with nail polish. Fluorescent images were acquired using the Zeiss LSM 510 Meta computerized image analysis system.

### Phylogenetic analysis

Phylogenetic trees were constructed using MEGA 5.0 software [59]. The amino acid sequences were aligned with Clustal X. The best-fit models of the trees were selected by ProtTest3.0 [60]. The branch length was calculated by the Maximum-Likelihood method with the PAM Matrix (Dayhoff) model (for peptide analysis combined with +I: invariable sites and +G: rate heterogeneity among sites; for receptor analysis combined with +I, +G and +F: observed amino acid frequencies) and reflected the estimated number of amino acid substitutions along each branch. 500 bootstrap simulations were used to test the reliability of branching. Numbers on the nodes of the trees indicated the percentage of bootstrap replicates in which the labelled branch was reproduced.

### Statistical analysis

Results are presented as mean ± SEM of duplicated assays in at least three independent experiments. GraphPad Prism version 5.0 (GraphPad Software, Inc., San Diego, CA) was used to plot the sigmoidal curves in the cAMP and calcium mobilization assays and to perform statistical analyses using one-way ANOVA followed by Dunnett's test. Differences were considered significant when p<0.01.

## Supporting Information

Figure S1 **Chromosomal locations of (A) PHI/VIP and (B) PRP/PACAP in various vertebrate species.** Genes adjacent to PHI/VIP and PRP/PACAP in different genomes are shown and linked to show their similarities in chromosomal location. The genes are named according to their annotation in the human genome. PHI/VIP and PRP/PACAP genes are boldfaced.
(PPTX)

Figure S2 **Chromosomal locations of (A) VPAC_1_, PAC_1_ and (B) VPAC_2_ in various vertebrate species.** Genes adjacent to VPAC_1_, PAC_1_ and VPAC_2_ in different genomes are shown and linked to show their similarities in chromosomal location. The genes are named according to their annotation in the human genome. VPAC_1_, PAC_1_ and VPAC_2_ genes are boldfaced.
(PPTX)

Figure S3 **Full length nucleotide and deduced amino acid sequences of Japanese lamprey (A) PHI/VIP and (B) PRP/PACAP cDNAs.** Numbers on the left correspond to the first nucleotide of each line. The nucleotide sequence has been translated into amino acid sequence according to the predicted signal peptide. The signal peptide sequences are highlighted in italics, mature peptide sequences highlighted in bold and the stop codon denoted by “*”.
(PPTX)

Figure S4 **Comparison of the amino acid sequences of the (A) PHI/VIP and (B) PRP/PACAP precursor peptides from various species as shown by amino acid sequence alignment.** The alignment was generated using the default settings of Vector NTI 10 (Invitrogen) with the AlignX program (Invitrogen). Residues have been highlighted as follows: identical (yellow), conserved (blue), similar (green). Residues have been highlighted as follows: identical (yellow), conserved (blue) and similar (green). Putative PHI and VIP peptides are boxed in red and processing sites are boxed in blue. The sequences contain a 16–30 amino acid long signal peptide (jlpPHI/VIP: 24-amino acid; jlpPRP/PACAP: 28-amino acid), one or more peptide hormone sequences (PHI and VIP; PRP and PACAP) and one or more spacer regions.
(PPTX)

Figure S5 **Full length nucleotide and deduced amino acid sequences of (A) jlpVPAC, (B) hfVPACa and (C) hfVPACb cDNAs.** Numbers on the left correspond to the first nucleotide of each line. The nucleotide sequence has been translated into amino acid sequence according to the predicted signal peptide. The signal peptide sequences are highlighted in italics, mature peptide sequences highlighted in bold and the stop codon denoted by “*”.
(PPTX)

Figure S6 **Comparison of the amino acid sequences of VIP/PACAP receptors from various species as shown by amino acid sequence alignment.** The alignment was generated using the default settings of Vector NTI 10 (Invitrogen) with the AlignX program (Invitrogen). Transmembrane (TM) domains are boxed in red and annotated; conserved motifs are boxed in blue. Conserved cysteine residues are denoted by “*” and N-glycosylation sites by “#”.
(PPTX)

Figure S7 **Percent amino acid homology of vertebrate (A) VPAC_1_, (B) VPAC_2_ and (C) PAC_1_ receptors.**
(PPTX)

Table S1 **List of primers used in PCR, real-time PCR and** ***in situ*** **hybridization.**
(PPTX)
