# Supplementary figures and images for: Agnathan VIP, PACAP and Their Receptors: Ancestral Origins of Today's Highly Diversified Forms
Source: PLoS One. 2012 Sep 5;7(9):e44691. doi: 10.1371/journal.pone.0044691 (PMC3434177; doi:10.1371/journal.pone.0044691)

## Slide 1
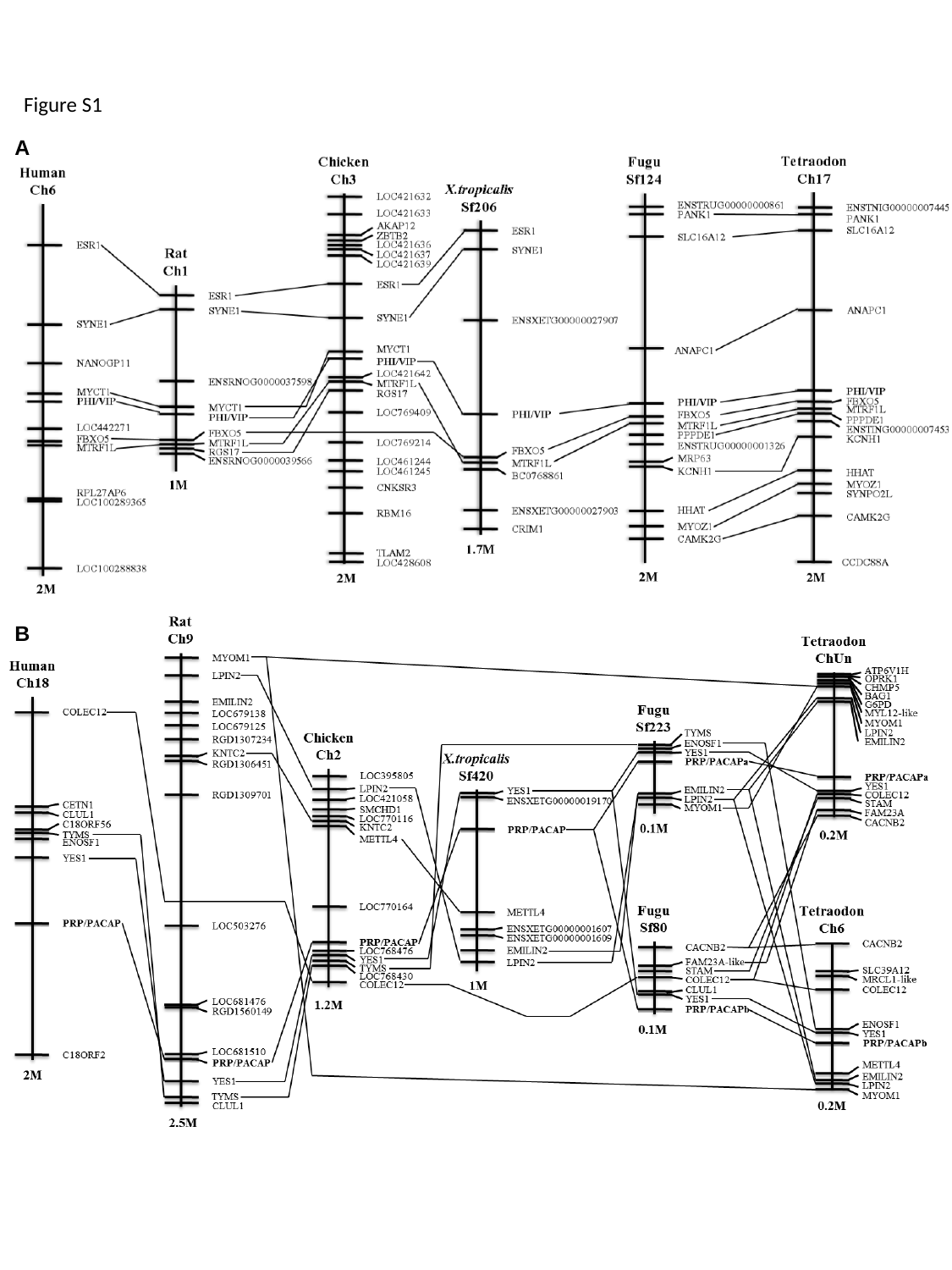

Figure S1
A
B

Supplement: Figure S1 — Chromosomal locations of (A) PHI/VIP and (B) PRP/PACAP in various vertebrate species. Genes adjacent to PHI/VIP and PRP/PACAP in different genomes are shown and linked to show their similarities in chromosomal location. The genes are named according to their annotation in the human genome. PHI/VIP and PRP/PACAP genes are boldfaced. (PPTX) [file pone.0044691.s001.pptx]

## Slide 1
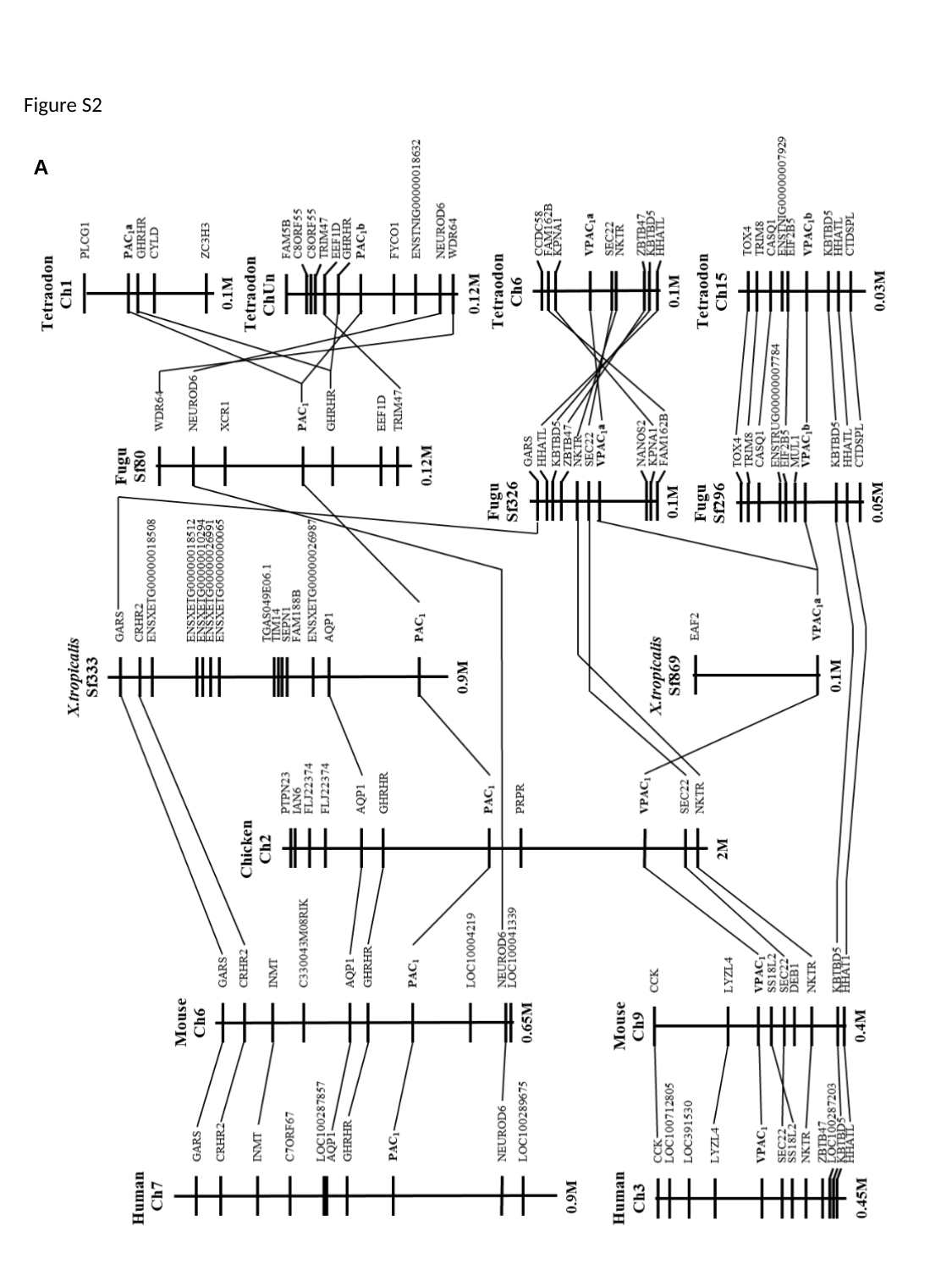

Figure S2
A

## Slide 2
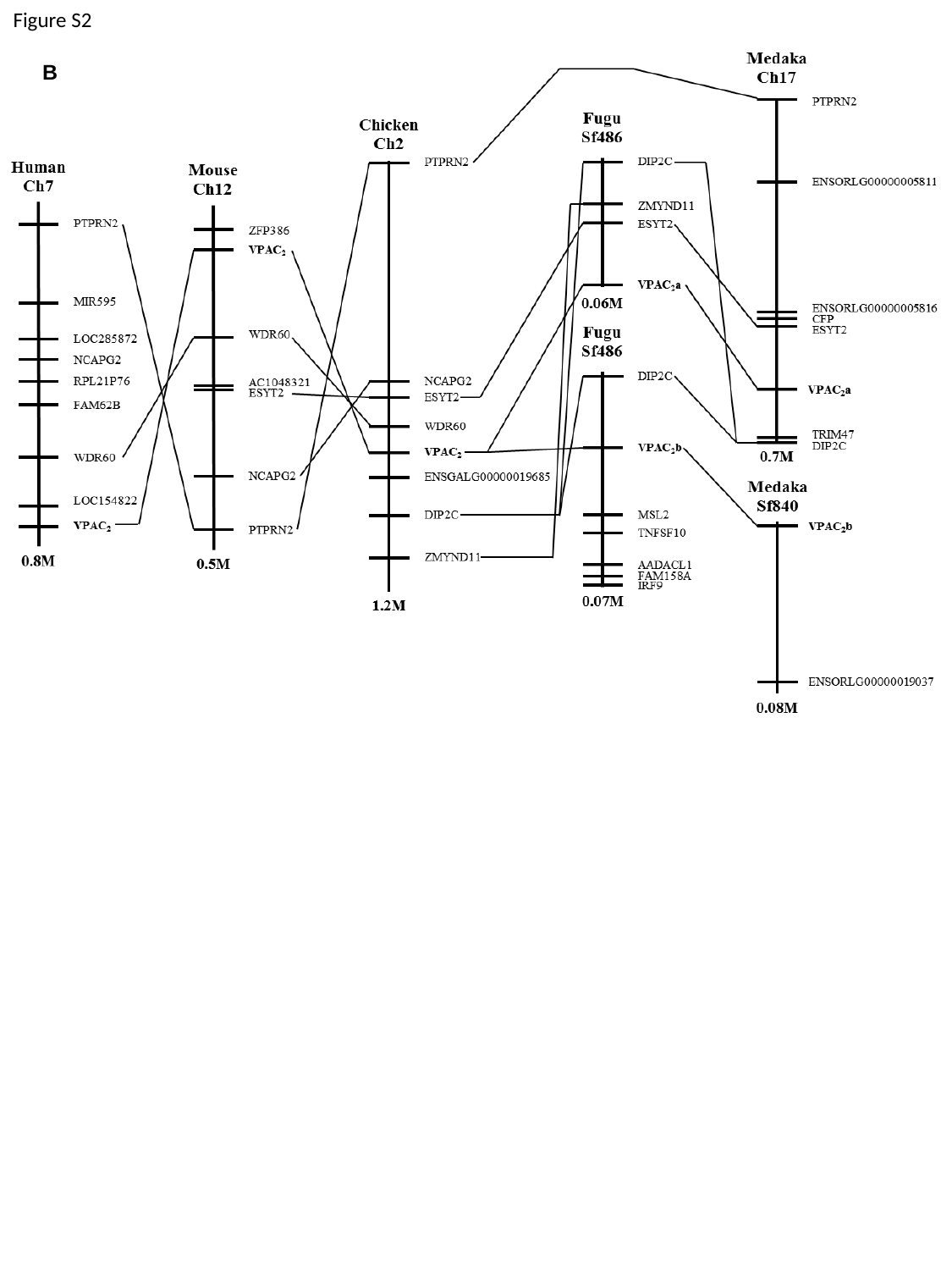

Figure S2
B

Supplement: Figure S2 — Chromosomal locations of (A) VPAC1, PAC1 and (B) VPAC2 in various vertebrate species. Genes adjacent to VPAC1, PAC1 and VPAC2 in different genomes are shown and linked to show their similarities in chromosomal location. The genes are named according to their annotation in the human genome. VPAC1, PAC1 and VPAC2 genes are boldfaced. (PPTX) [file pone.0044691.s002.pptx]

## Slide 1
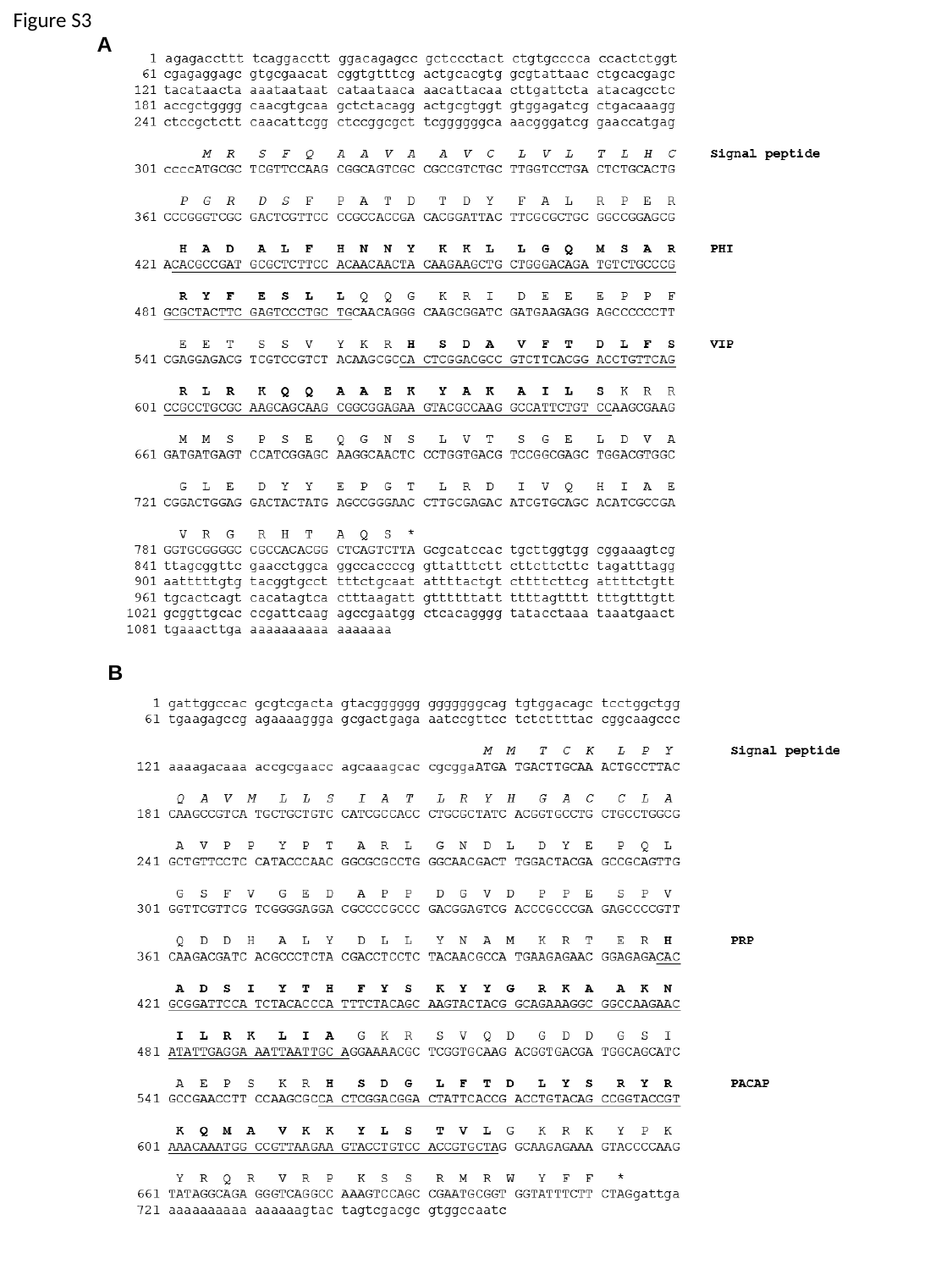

Figure S3
A
B

Supplement: Figure S3 — Full length nucleotide and deduced amino acid sequences of Japanese lamprey (A) PHI/VIP and (B) PRP/PACAP cDNAs. Numbers on the left correspond to the first nucleotide of each line. The nucleotide sequence has been translated into amino acid sequence according to the predicted signal peptide. The signal peptide sequences are highlighted in italics, mature peptide sequences highlighted in bold and the stop codon denoted by “*”. (PPTX) [file pone.0044691.s003.pptx]

## Slide 1
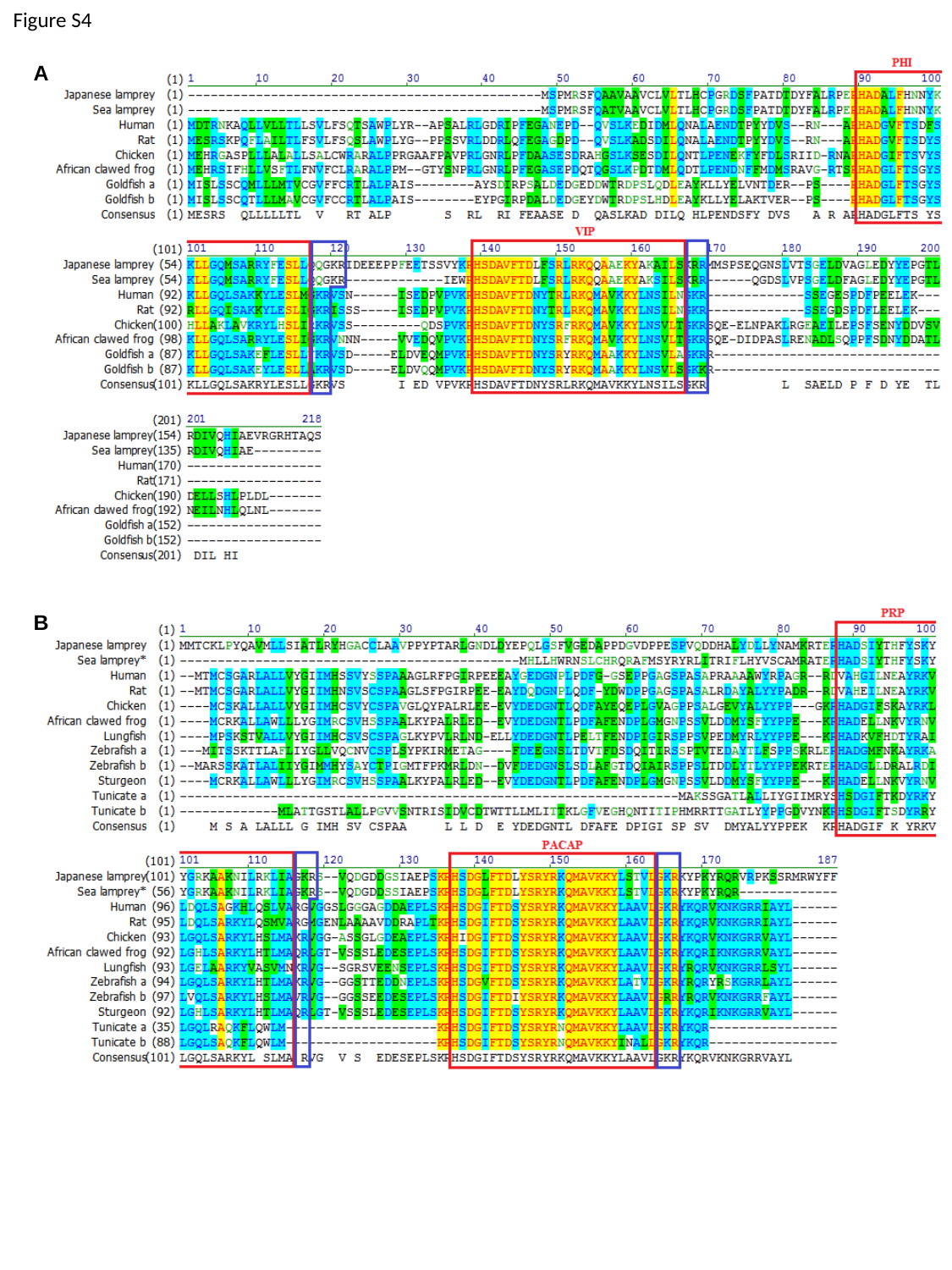

Figure S4
A
B

Supplement: Figure S4 — Comparison of the amino acid sequences of the (A) PHI/VIP and (B) PRP/PACAP precursor peptides from various species as shown by amino acid sequence alignment. The alignment was generated using the default settings of Vector NTI 10 (Invitrogen) with the AlignX program (Invitrogen). Residues have been highlighted as follows: identical (yellow), conserved (blue), similar (green). Residues have been highlighted as follows: identical (yellow), conserved (blue) and similar (green). Putative PHI and VIP peptides are boxed in red and processing sites are boxed in blue. The sequences contain a 16–30 amino acid long signal peptide (jlpPHI/VIP: 24-amino acid; jlpPRP/PACAP: 28-amino acid), one or more peptide hormone sequences (PHI and VIP; PRP and PACAP) and one or more spacer regions. (PPTX) [file pone.0044691.s004.pptx]

## Slide 1
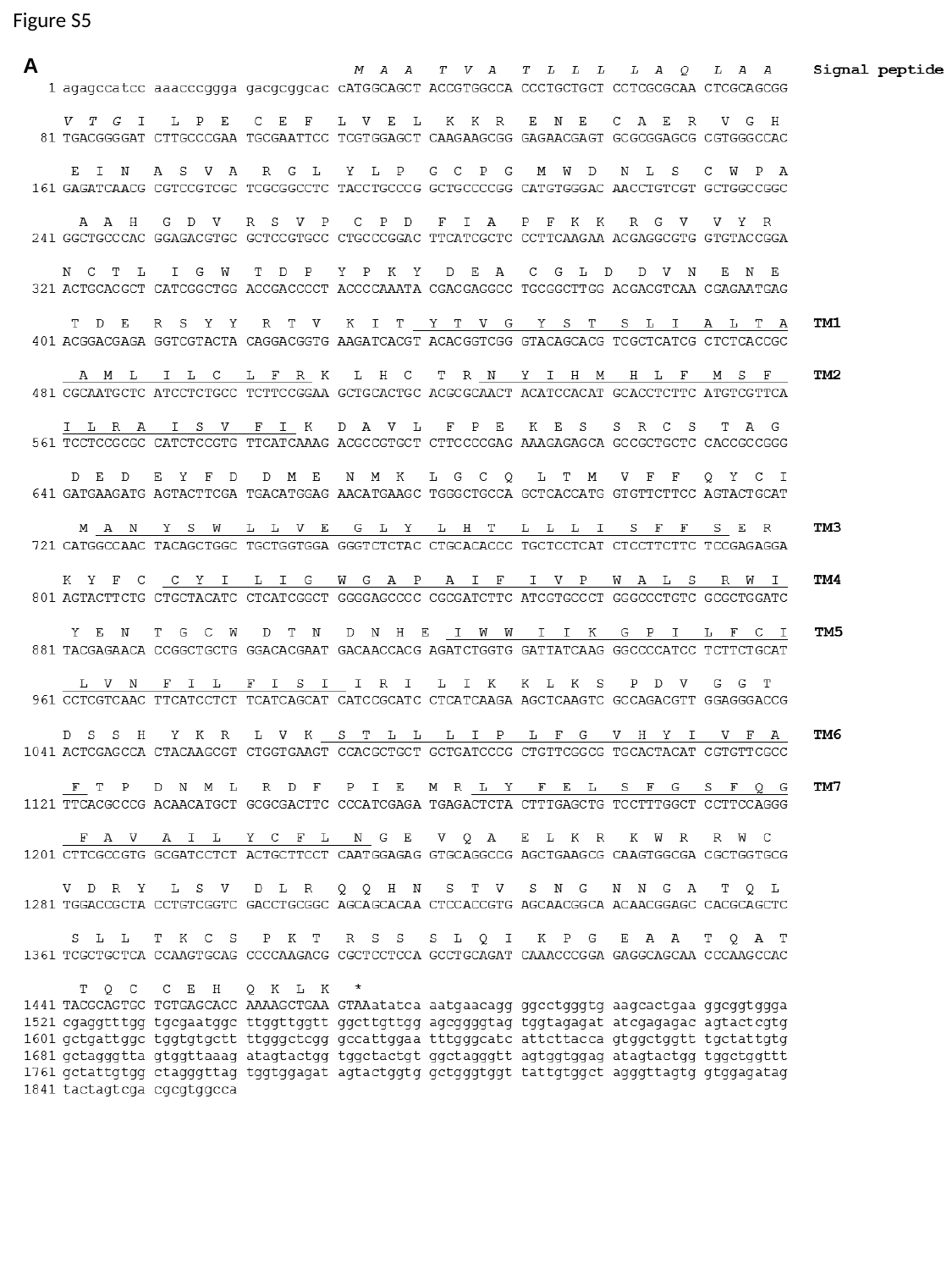

Figure S5
A

## Slide 2
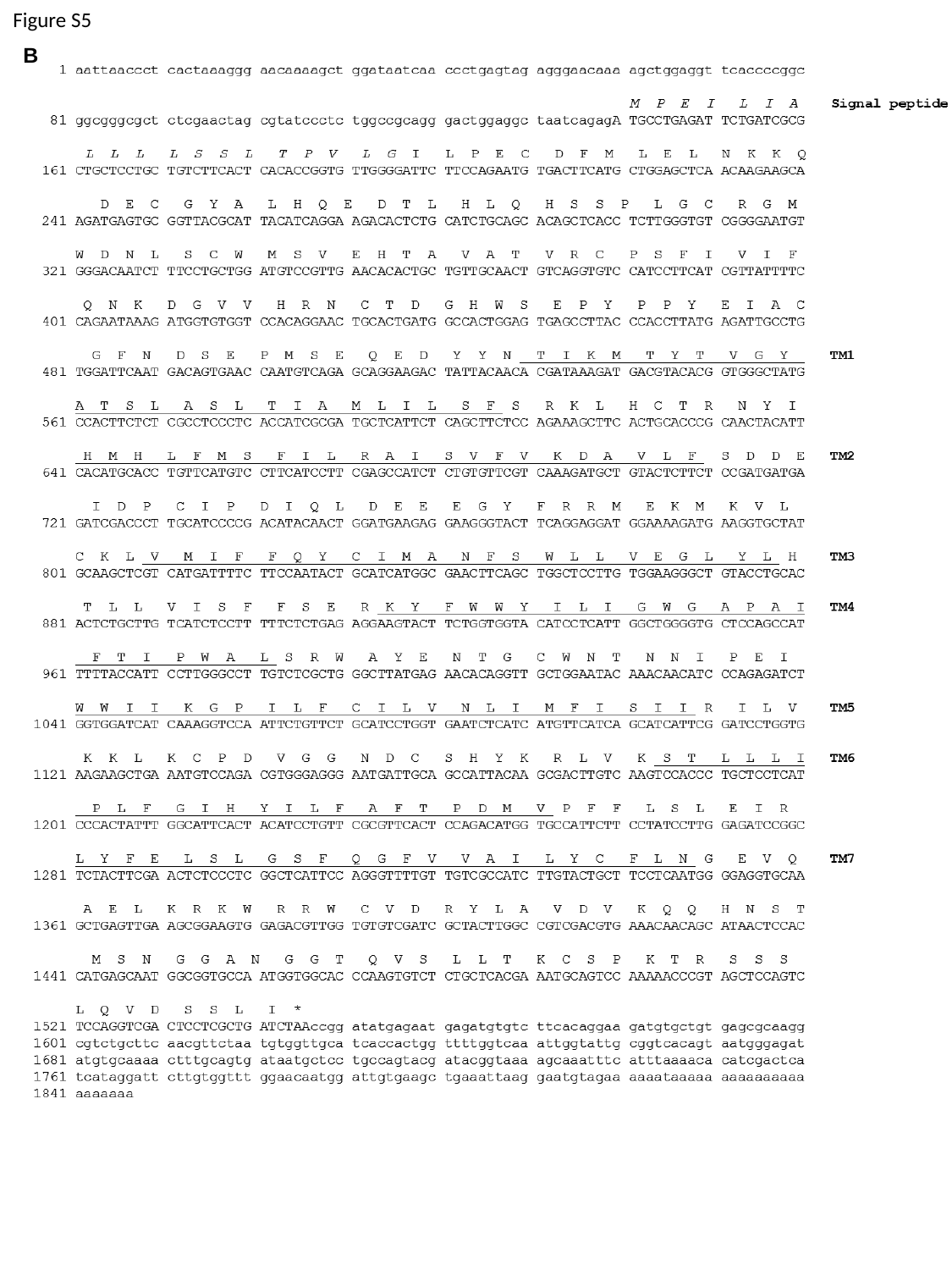

Figure S5
B

## Slide 3
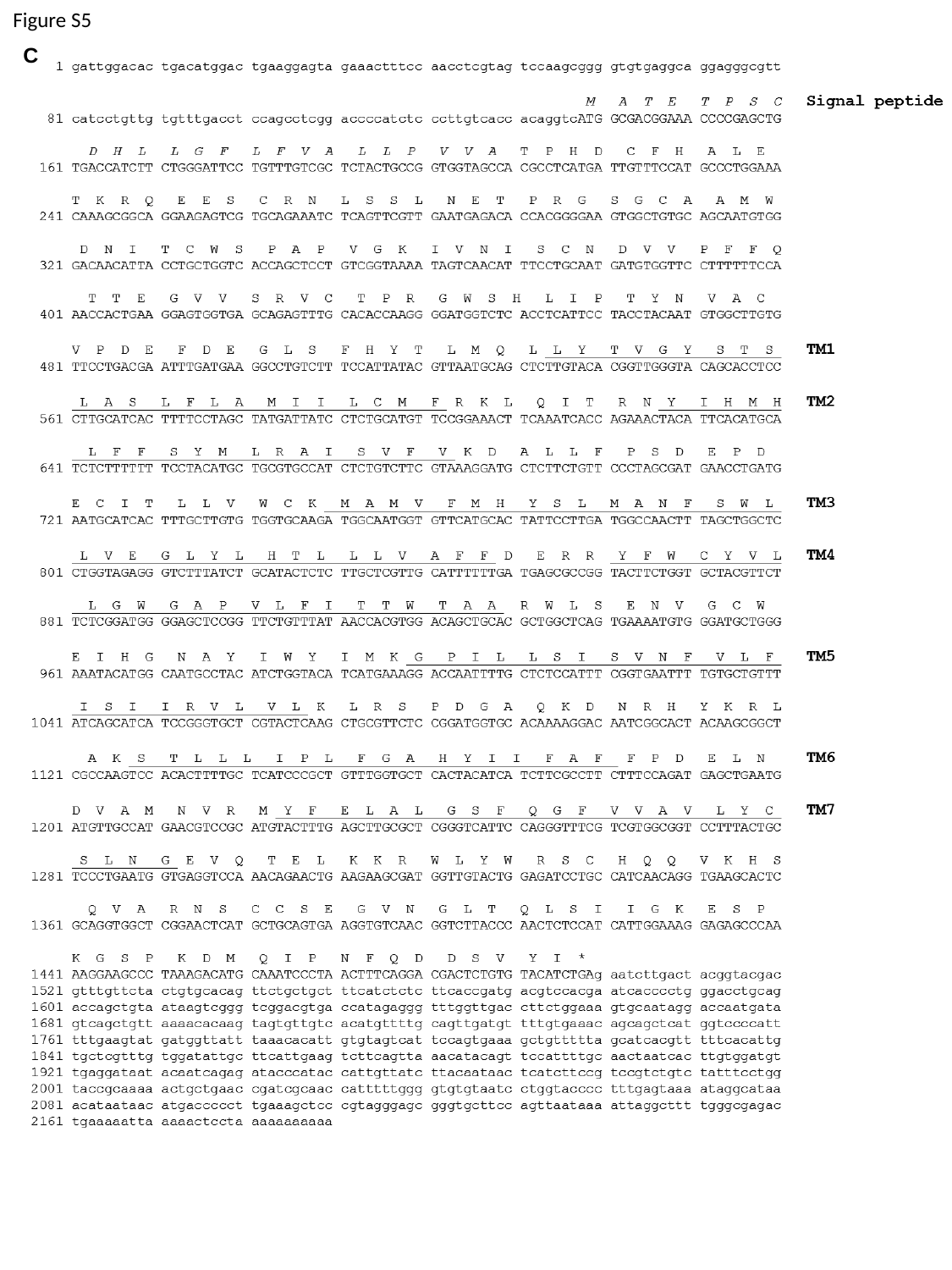

Figure S5
C

Supplement: Figure S5 — Full length nucleotide and deduced amino acid sequences of (A) jlpVPAC, (B) hfVPACa and (C) hfVPACb cDNAs. Numbers on the left correspond to the first nucleotide of each line. The nucleotide sequence has been translated into amino acid sequence according to the predicted signal peptide. The signal peptide sequences are highlighted in italics, mature peptide sequences highlighted in bold and the stop codon denoted by “*”. (PPTX) [file pone.0044691.s005.pptx]

## Slide 1
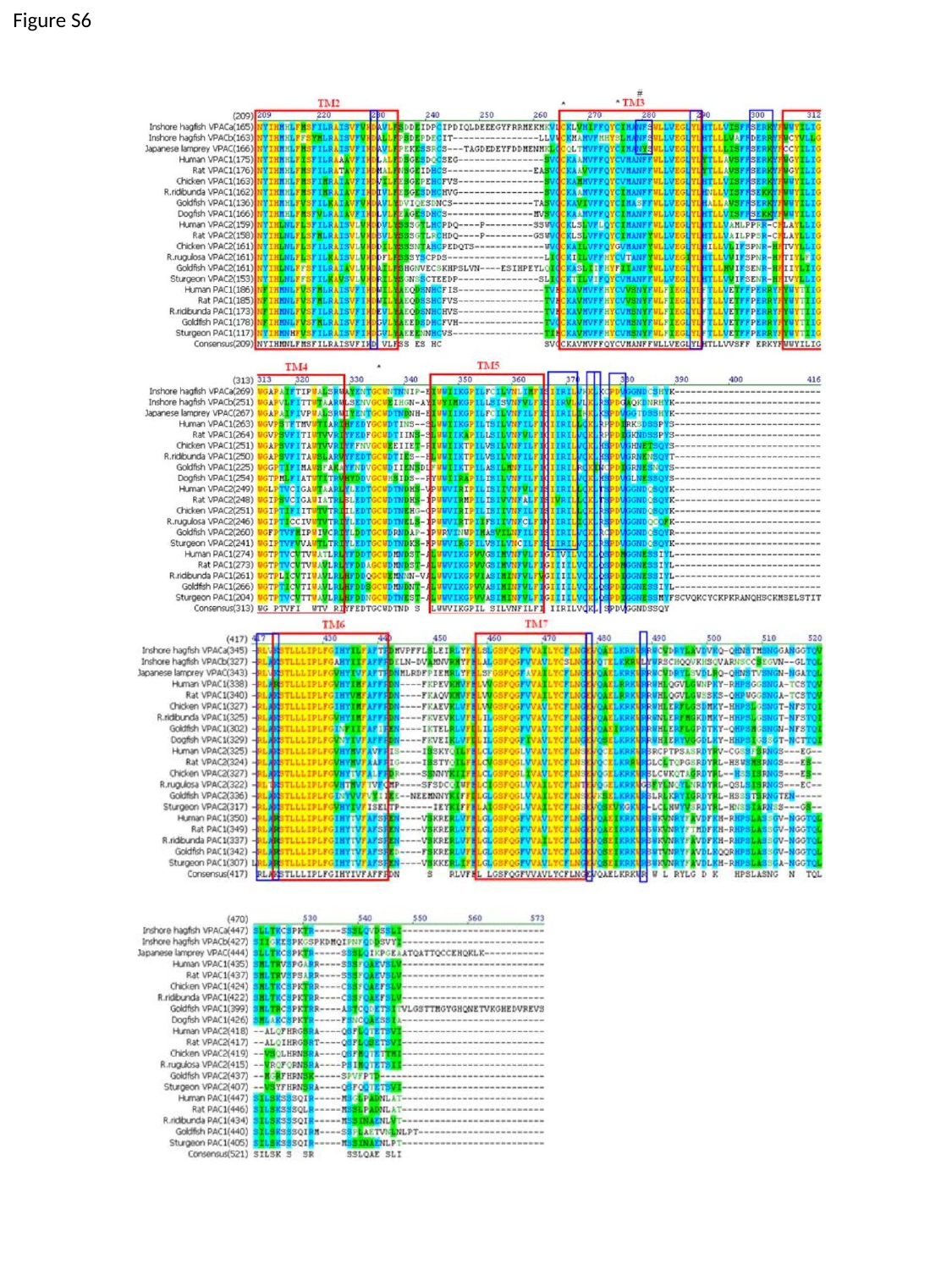

Figure S6

Supplement: Figure S6 — Comparison of the amino acid sequences of VIP/PACAP receptors from various species as shown by amino acid sequence alignment. The alignment was generated using the default settings of Vector NTI 10 (Invitrogen) with the AlignX program (Invitrogen). Transmembrane (TM) domains are boxed in red and annotated; conserved motifs are boxed in blue. Conserved cysteine residues are denoted by “*” and N-glycosylation sites by “#”. (PPTX) [file pone.0044691.s006.pptx]
