# Supplementary material for: Agnathan VIP, PACAP and Their Receptors: Ancestral Origins of Today's Highly Diversified Forms
Source: PLoS One. 2012 Sep 5;7(9):e44691. doi: 10.1371/journal.pone.0044691 (PMC3434177; doi:10.1371/journal.pone.0044691)
Supplement: Figure S7 — Percent amino acid homology of vertebrate (A) VPAC1, (B) VPAC2 and (C) PAC1 receptors. (PPTX) [file pone.0044691.s007.pptx]

## Slide 1
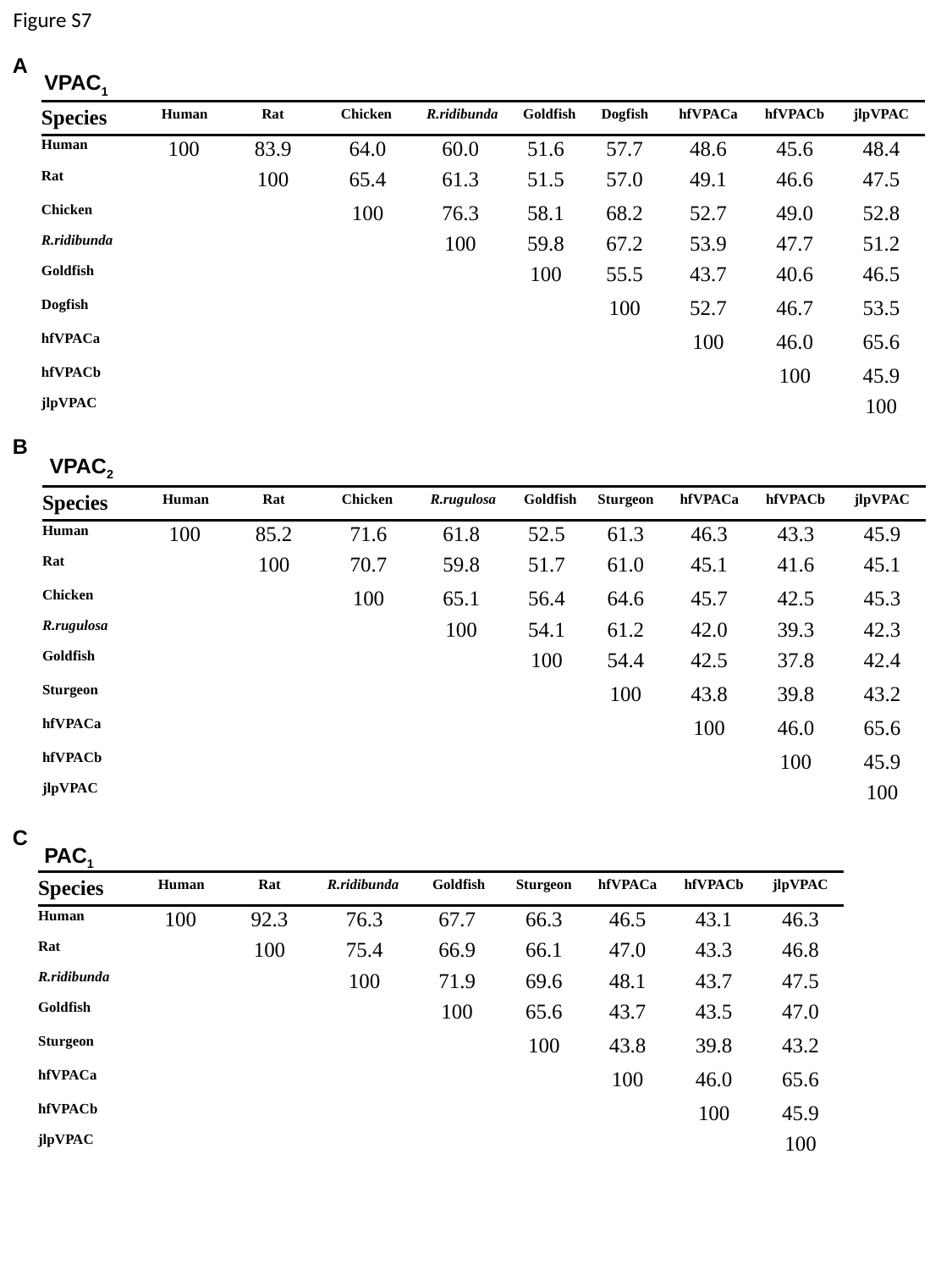

Figure S7
A
VPAC1
| Species | Human | | Rat | Chicken | R.ridibunda | Goldfish | Dogfish | hfVPACa | hfVPACb | jlpVPAC |
| --- | --- | --- | --- | --- | --- | --- | --- | --- | --- | --- |
| Human | | 100 | 83.9 | 64.0 | 60.0 | 51.6 | 57.7 | 48.6 | 45.6 | 48.4 |
| Rat | | | 100 | 65.4 | 61.3 | 51.5 | 57.0 | 49.1 | 46.6 | 47.5 |
| Chicken | | | | 100 | 76.3 | 58.1 | 68.2 | 52.7 | 49.0 | 52.8 |
| R.ridibunda | | | | | 100 | 59.8 | 67.2 | 53.9 | 47.7 | 51.2 |
| Goldfish | | | | | | 100 | 55.5 | 43.7 | 40.6 | 46.5 |
| Dogfish | | | | | | | 100 | 52.7 | 46.7 | 53.5 |
| hfVPACa | | | | | | | | 100 | 46.0 | 65.6 |
| hfVPACb | | | | | | | | | 100 | 45.9 |
| jlpVPAC | | | | | | | | | | 100 |
B
VPAC2
| Species | Human | | Rat | Chicken | R.rugulosa | Goldfish | Sturgeon | hfVPACa | hfVPACb | jlpVPAC |
| --- | --- | --- | --- | --- | --- | --- | --- | --- | --- | --- |
| Human | | 100 | 85.2 | 71.6 | 61.8 | 52.5 | 61.3 | 46.3 | 43.3 | 45.9 |
| Rat | | | 100 | 70.7 | 59.8 | 51.7 | 61.0 | 45.1 | 41.6 | 45.1 |
| Chicken | | | | 100 | 65.1 | 56.4 | 64.6 | 45.7 | 42.5 | 45.3 |
| R.rugulosa | | | | | 100 | 54.1 | 61.2 | 42.0 | 39.3 | 42.3 |
| Goldfish | | | | | | 100 | 54.4 | 42.5 | 37.8 | 42.4 |
| Sturgeon | | | | | | | 100 | 43.8 | 39.8 | 43.2 |
| hfVPACa | | | | | | | | 100 | 46.0 | 65.6 |
| hfVPACb | | | | | | | | | 100 | 45.9 |
| jlpVPAC | | | | | | | | | | 100 |
C
PAC1
| Species | Human | | Rat | R.ridibunda | Goldfish | Sturgeon | hfVPACa | hfVPACb | jlpVPAC |
| --- | --- | --- | --- | --- | --- | --- | --- | --- | --- |
| Human | | 100 | 92.3 | 76.3 | 67.7 | 66.3 | 46.5 | 43.1 | 46.3 |
| Rat | | | 100 | 75.4 | 66.9 | 66.1 | 47.0 | 43.3 | 46.8 |
| R.ridibunda | | | | 100 | 71.9 | 69.6 | 48.1 | 43.7 | 47.5 |
| Goldfish | | | | | 100 | 65.6 | 43.7 | 43.5 | 47.0 |
| Sturgeon | | | | | | 100 | 43.8 | 39.8 | 43.2 |
| hfVPACa | | | | | | | 100 | 46.0 | 65.6 |
| hfVPACb | | | | | | | | 100 | 45.9 |
| jlpVPAC | | | | | | | | | 100 |
