# Supplementary material for: Agnathan VIP, PACAP and Their Receptors: Ancestral Origins of Today's Highly Diversified Forms
Source: PLoS One. 2012 Sep 5;7(9):e44691. doi: 10.1371/journal.pone.0044691 (PMC3434177; doi:10.1371/journal.pone.0044691)
Supplement: Table S1 — List of primers used in PCR, real-time PCR and in situ hybridization. (PPTX) [file pone.0044691.s008.pptx]

## Slide 1
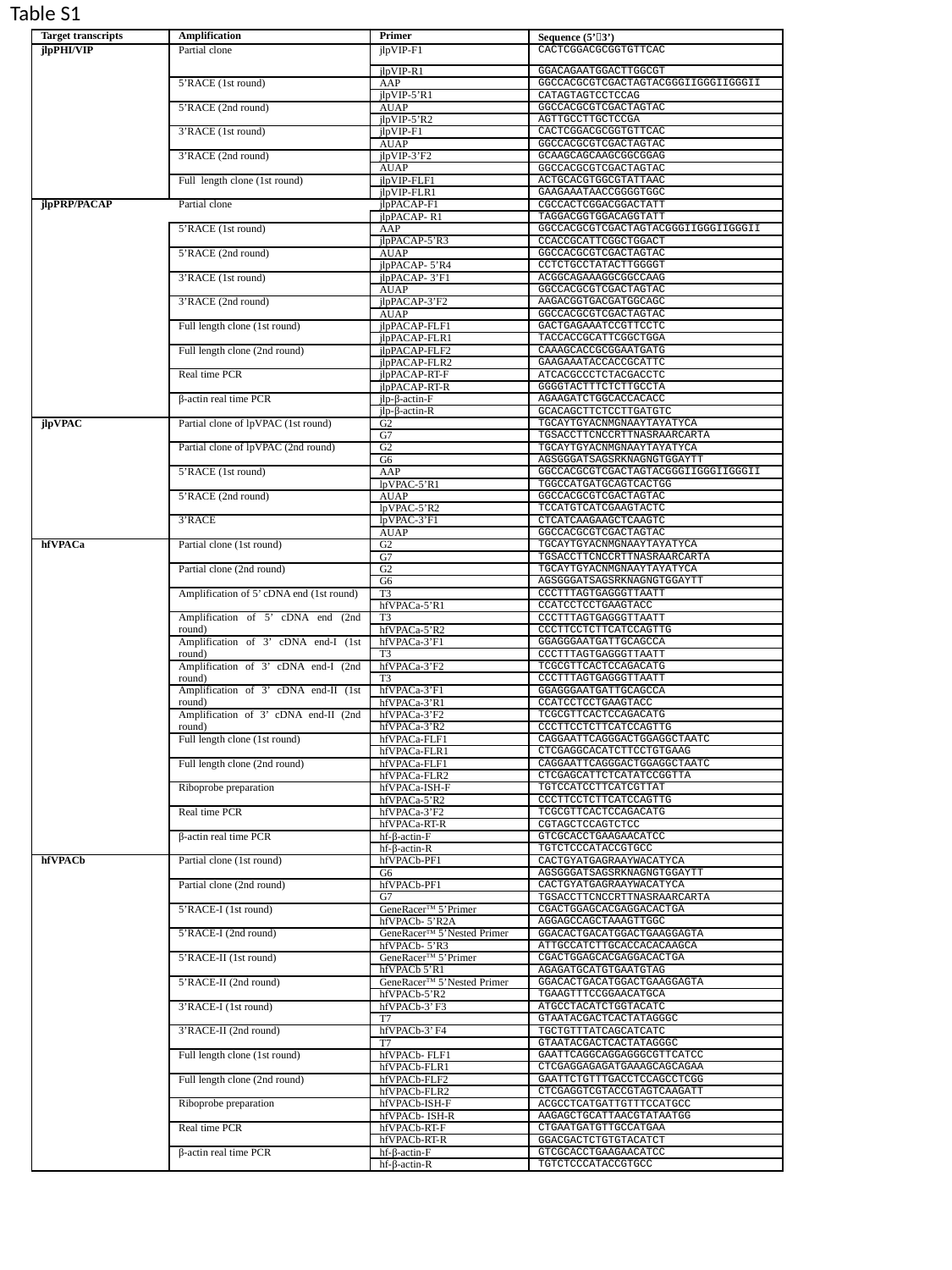

Table S1
| Target transcripts | Amplification | Primer | Sequence (5’3’) |
| --- | --- | --- | --- |
| jlpPHI/VIP | Partial clone | jlpVIP-F1 | CACTCGGACGCGGTGTTCAC |
| | | jlpVIP-R1 | GGACAGAATGGACTTGGCGT |
| | 5’RACE (1st round) | AAP | GGCCACGCGTCGACTAGTACGGGIIGGGIIGGGII |
| | | jlpVIP-5’R1 | CATAGTAGTCCTCCAG |
| | 5’RACE (2nd round) | AUAP | GGCCACGCGTCGACTAGTAC |
| | | jlpVIP-5’R2 | AGTTGCCTTGCTCCGA |
| | 3’RACE (1st round) | jlpVIP-F1 | CACTCGGACGCGGTGTTCAC |
| | | AUAP | GGCCACGCGTCGACTAGTAC |
| | 3’RACE (2nd round) | jlpVIP-3’F2 | GCAAGCAGCAAGCGGCGGAG |
| | | AUAP | GGCCACGCGTCGACTAGTAC |
| | Full length clone (1st round) | jlpVIP-FLF1 | ACTGCACGTGGCGTATTAAC |
| | | jlpVIP-FLR1 | GAAGAAATAACCGGGGTGGC |
| jlpPRP/PACAP | Partial clone | jlpPACAP-F1 | CGCCACTCGGACGGACTATT |
| | | jlpPACAP- R1 | TAGGACGGTGGACAGGTATT |
| | 5’RACE (1st round) | AAP | GGCCACGCGTCGACTAGTACGGGIIGGGIIGGGII |
| | | jlpPACAP-5’R3 | CCACCGCATTCGGCTGGACT |
| | 5’RACE (2nd round) | AUAP | GGCCACGCGTCGACTAGTAC |
| | | jlpPACAP- 5’R4 | CCTCTGCCTATACTTGGGGT |
| | 3’RACE (1st round) | jlpPACAP- 3’F1 | ACGGCAGAAAGGCGGCCAAG |
| | | AUAP | GGCCACGCGTCGACTAGTAC |
| | 3’RACE (2nd round) | jlpPACAP-3’F2 | AAGACGGTGACGATGGCAGC |
| | | AUAP | GGCCACGCGTCGACTAGTAC |
| | Full length clone (1st round) | jlpPACAP-FLF1 | GACTGAGAAATCCGTTCCTC |
| | | jlpPACAP-FLR1 | TACCACCGCATTCGGCTGGA |
| | Full length clone (2nd round) | jlpPACAP-FLF2 | CAAAGCACCGCGGAATGATG |
| | | jlpPACAP-FLR2 | GAAGAAATACCACCGCATTC |
| | Real time PCR | jlpPACAP-RT-F | ATCACGCCCTCTACGACCTC |
| | | jlpPACAP-RT-R | GGGGTACTTTCTCTTGCCTA |
| | β-actin real time PCR | jlp-β-actin-F | AGAAGATCTGGCACCACACC |
| | | jlp-β-actin-R | GCACAGCTTCTCCTTGATGTC |
| jlpVPAC | Partial clone of lpVPAC (1st round) | G2 | TGCAYTGYACNMGNAAYTAYATYCA |
| | | G7 | TGSACCTTCNCCRTTNASRAARCARTA |
| | Partial clone of lpVPAC (2nd round) | G2 | TGCAYTGYACNMGNAAYTAYATYCA |
| | | G6 | AGSGGGATSAGSRKNAGNGTGGAYTT |
| | 5’RACE (1st round) | AAP | GGCCACGCGTCGACTAGTACGGGIIGGGIIGGGII |
| | | lpVPAC-5’R1 | TGGCCATGATGCAGTCACTGG |
| | 5’RACE (2nd round) | AUAP | GGCCACGCGTCGACTAGTAC |
| | | lpVPAC-5’R2 | TCCATGTCATCGAAGTACTC |
| | 3’RACE | lpVPAC-3’F1 | CTCATCAAGAAGCTCAAGTC |
| | | AUAP | GGCCACGCGTCGACTAGTAC |
| hfVPACa | Partial clone (1st round) | G2 | TGCAYTGYACNMGNAAYTAYATYCA |
| | | G7 | TGSACCTTCNCCRTTNASRAARCARTA |
| | Partial clone (2nd round) | G2 | TGCAYTGYACNMGNAAYTAYATYCA |
| | | G6 | AGSGGGATSAGSRKNAGNGTGGAYTT |
| | Amplification of 5’ cDNA end (1st round) | T3 | CCCTTTAGTGAGGGTTAATT |
| | | hfVPACa-5’R1 | CCATCCTCCTGAAGTACC |
| | Amplification of 5’ cDNA end (2nd round) | T3 | CCCTTTAGTGAGGGTTAATT |
| | | hfVPACa-5’R2 | CCCTTCCTCTTCATCCAGTTG |
| | Amplification of 3’ cDNA end-I (1st round) | hfVPACa-3’F1 | GGAGGGAATGATTGCAGCCA |
| | | T3 | CCCTTTAGTGAGGGTTAATT |
| | Amplification of 3’ cDNA end-I (2nd round) | hfVPACa-3’F2 | TCGCGTTCACTCCAGACATG |
| | | T3 | CCCTTTAGTGAGGGTTAATT |
| | Amplification of 3’ cDNA end-II (1st round) | hfVPACa-3’F1 | GGAGGGAATGATTGCAGCCA |
| | | hfVPACa-3’R1 | CCATCCTCCTGAAGTACC |
| | Amplification of 3’ cDNA end-II (2nd round) | hfVPACa-3’F2 | TCGCGTTCACTCCAGACATG |
| | | hfVPACa-3’R2 | CCCTTCCTCTTCATCCAGTTG |
| | Full length clone (1st round) | hfVPACa-FLF1 | CAGGAATTCAGGGACTGGAGGCTAATC |
| | | hfVPACa-FLR1 | CTCGAGGCACATCTTCCTGTGAAG |
| | Full length clone (2nd round) | hfVPACa-FLF1 | CAGGAATTCAGGGACTGGAGGCTAATC |
| | | hfVPACa-FLR2 | CTCGAGCATTCTCATATCCGGTTA |
| | Riboprobe preparation | hfVPACa-ISH-F | TGTCCATCCTTCATCGTTAT |
| | | hfVPACa-5’R2 | CCCTTCCTCTTCATCCAGTTG |
| | Real time PCR | hfVPACa-3’F2 | TCGCGTTCACTCCAGACATG |
| | | hfVPACa-RT-R | CGTAGCTCCAGTCTCC |
| | β-actin real time PCR | hf-β-actin-F | GTCGCACCTGAAGAACATCC |
| | | hf-β-actin-R | TGTCTCCCATACCGTGCC |
| hfVPACb | Partial clone (1st round) | hfVPACb-PF1 | CACTGYATGAGRAAYWACATYCA |
| | | G6 | AGSGGGATSAGSRKNAGNGTGGAYTT |
| | Partial clone (2nd round) | hfVPACb-PF1 | CACTGYATGAGRAAYWACATYCA |
| | | G7 | TGSACCTTCNCCRTTNASRAARCARTA |
| | 5’RACE-I (1st round) | GeneRacerTM 5’Primer | CGACTGGAGCACGAGGACACTGA |
| | | hfVPACb- 5’R2A | AGGAGCCAGCTAAAGTTGGC |
| | 5’RACE-I (2nd round) | GeneRacerTM 5’Nested Primer | GGACACTGACATGGACTGAAGGAGTA |
| | | hfVPACb- 5’R3 | ATTGCCATCTTGCACCACACAAGCA |
| | 5’RACE-II (1st round) | GeneRacerTM 5’Primer | CGACTGGAGCACGAGGACACTGA |
| | | hfVPACb 5’R1 | AGAGATGCATGTGAATGTAG |
| | 5’RACE-II (2nd round) | GeneRacerTM 5’Nested Primer | GGACACTGACATGGACTGAAGGAGTA |
| | | hfVPACb-5’R2 | TGAAGTTTCCGGAACATGCA |
| | 3’RACE-I (1st round) | hfVPACb-3’ F3 | ATGCCTACATCTGGTACATC |
| | | T7 | GTAATACGACTCACTATAGGGC |
| | 3’RACE-II (2nd round) | hfVPACb-3’ F4 | TGCTGTTTATCAGCATCATC |
| | | T7 | GTAATACGACTCACTATAGGGC |
| | Full length clone (1st round) | hfVPACb- FLF1 | GAATTCAGGCAGGAGGGCGTTCATCC |
| | | hfVPACb-FLR1 | CTCGAGGAGAGATGAAAGCAGCAGAA |
| | Full length clone (2nd round) | hfVPACb-FLF2 | GAATTCTGTTTGACCTCCAGCCTCGG |
| | | hfVPACb-FLR2 | CTCGAGGTCGTACCGTAGTCAAGATT |
| | Riboprobe preparation | hfVPACb-ISH-F | ACGCCTCATGATTGTTTCCATGCC |
| | | hfVPACb- ISH-R | AAGAGCTGCATTAACGTATAATGG |
| | Real time PCR | hfVPACb-RT-F | CTGAATGATGTTGCCATGAA |
| | | hfVPACb-RT-R | GGACGACTCTGTGTACATCT |
| | β-actin real time PCR | hf-β-actin-F | GTCGCACCTGAAGAACATCC |
| | | hf-β-actin-R | TGTCTCCCATACCGTGCC |
